# Silk Web Removal Enhances the Efficacy of *Beauveria bassiana* Treatment Against *Hyphantria cunea*: Insights from Immune and Physiological Parameters

**DOI:** 10.3390/insects17101039

**Published:** 2026-10-10

**Authors:** Hong Jiang, Peng Li, Ying Wang, Ming-Tao Tan, Ao-Ying Zhang, Shan-Chun Yan, Dun Jiang

**Affiliations:** 1 School of Forestry, Northeast Forestry University, Harbin 150040, China; 2 Key Laboratory of Sustainable Forest Ecosystem Management-Ministry of Education, Northeast Forestry University, Harbin 150040, China; 3 Heilongjiang Academy of Agricultural Reclamation Sciences, Harbin 150038, China

**Keywords:** silk web, conidial exposure, physical shelter, entomopathogenic fungi, biological control, *Hyphantria cunea*

## Abstract

The construction of physical shelters represents a widespread survival strategy among insects. This study evaluated the effect of silk web removal on the efficacy of *Beauveria bassiana* treatment against *Hyphantria cunea* larvae. Results indicated that the removal of silk webs significantly increased larval mortality and enhanced conidia attachment to the larval body during *B. bassiana* infection. Removal of the silk web increased larval mortality from 23.06 ± 3.14% to 46.94 ± 3.20%, with the presence of the silk web thus corresponding to a 50.88% relative reduction in *B. bassiana*-induced mortality. Scanning electron microscopy showed that conidial deposition on the larval cuticle was 2.8-fold higher, and markedly more aggregated within cuticular folds, in web-removed larvae, indicating that the silk web substantially lowers the conidial exposure actually experienced by the larvae. This difference in conidial exposure between silk web-bearing and silk-web-removed larvae was accompanied by multi-level physiological responses in the host. Silk web removal decreased the cellular immune response of *H. cunea* larvae to *B. bassiana*, as evidenced by reduced hemocyte counts, lower encapsulation and melanization activity, and altered expression of cellular immune-related genes (e.g., *Galectin-1* and *PPO2*). Silk web removal intensified the oxidative damage manifested by MDA and H_2_O_2_ and the cell apoptosis manifested by apoptosis regulatory genes (e.g., *AIFM3* and *Metacaspase-1-like*), accompanied by a significant decrease in the antioxidant enzyme activity and total antioxidant capacity in *B. bassiana*-infected larvae. The depletion of nutrient reserves (e.g., glucose) by *B. bassiana* from the *H. cunea* larvae was exacerbated upon silk web removal. Moreover, larvae exhibited a heightened response to *B. bassiana* infection upon silk web removal, including upregulated expression of cuticular protein genes and humoral immunity regulators (e.g., *Lebocin* and *Attacin*), antioxidant genes, and key enzymes in the glycolytic and tricarboxylic acid cycles. Collectively, the silk web reduces the efficacy of *B. bassiana* treatment against *H. cunea* larvae; this reduction is accompanied by a markedly lower conidial load on the cuticle, together with changes in the host’s immune defense, oxidative stress, and energy metabolism.

## Simple Summary

The silk web acts as a physical barrier that intercepts conidial suspension, thereby diminishing the efficacy of *Beauveria bassiana* against *Hyphantria cunea* larvae. Larvae from which the silk web had been removed carried substantially more conidia on the cuticle and died at more than twice the rate of silk web larvae. Upon removal of the silk web, *H. cunea* larvae likely activate cuticular defenses to counteract *B. bassiana* invasion. This was accompanied by a significant decline in cellular immunity against the infection, necessitating the activation of humoral immunity to resist the pathogen. The oxidative stress response in *H. cunea* larvae, triggered by *B. bassiana*, is likewise intensified following silk web removal, with greater oxidative damage and apoptosis. Scanning electron microscopy showed that silk-web-removed larvae carried a 2.8-fold higher conidial load on the cuticle; this higher conidial exposure is a plausible major contributor to the differential responses described above. It is worth noting that no significant differences in any measured parameter were detected between larvae that experienced silk web removal and those that did not in the absence of fungal challenge, which largely excludes interference from silk web removal handling or web reconstruction with the above conclusions.

## 1. Introduction

Global climate change has exacerbated the prevalence and impact of pests, leading to increasingly severe agricultural damage [1,2]. The Food and Agriculture Organization of the United Nations (FAO) estimates that pests are responsible for annual crop yield losses ranging from 10% to 28% worldwide. Phytophagous insects, in particular, account for a significant portion of these losses, with economic damages reaching up to 100 billion dollars [3]. These insects, which constitute approximately 40–50% of all insect species [4], are highly diverse and widely distributed. With the expansion of international trade, the introduction of invasive alien pests into new regions is increasing [5]. Favorable environmental conditions and the absence of natural predators enable these pests to spread and establish themselves in non-native habitats [6]. Invasive species have thus emerged as a critical threat to both agriculture and forestry [7,8]. In China, 21 species of invasive pests are prioritized for management, contributing to an annual economic loss in agriculture and forestry of up to 54.7 billion yuan [9].

A range of control methods, including chemical, physical, and biological strategies, have been developed to maintain ecological balance and enhance agricultural and forestry productivity [10,11,12]. While chemical control has traditionally been the dominant approach, its excessive use has led to issues such as pest resistance, pesticide residues, and pest resurgence [13]. In contrast, biological control utilizing entomopathogenic microorganisms offers several advantages, including minimal environmental impact, low toxicity to non-target species, and long-term efficacy [14]. Among these microorganisms, entomopathogenic fungi (EPF) represent a key biocontrol agent, increasingly recognized for their potential to substitute chemical insecticides in pest management [15,16]. Statistical data indicate that fungal infections account for approximately 60% of insect mortality caused by pathogenic microorganisms [17,18]. *Beauveria bassiana* and *Metarhizium* sp. have emerged as the primary fungal species utilized as biocontrol agents. For instance, *B. bassiana* is effective in managing significant agricultural and forestry pests, such as *Ostrinia furnacalis* [19], *Dendrolimus punctatus* [20], *Helicoverpa armigera* [21], and *Spodoptera frugiperda* [22]. In practical applications, the insecticidal efficacy of EPF is frequently influenced by a range of interacting, multifaceted factors [23]. Genetic traits of fungal strains represent a key internal determinant of their pathogenic potential. Furthermore, the insect host’s physical barriers and complex innate immune defenses mitigate fungal pathogenicity [24]. Environmental factors, including temperature, humidity, and ultraviolet radiation, also play a critical role in modulating the virulence of EPF against insect hosts [25].

The construction of physical shelters is a prevalent survival strategy among insects. Species exhibiting collective behavior or producing silk, such as certain pests, are particularly adept at creating such shelters. Examples include Lepidopteran species like *Haritalodes derogata* [26], and *H. armigera* [27], as well as Hymenopterans such as *Apis cerana* [28], *Pheidole megacephala* [29], and Trichoptera like *Caddisflies* [30]. The timing and methods of shelter construction vary across species. Lepidopteran larvae typically construct shelters by spinning silk around leaves or binding surrounding foliage to form cocoons or webs. Physical shelters serve as vital defensive mechanisms against natural predators. For instance, *H. armigera* constructs silk cocoons between plant leaves or on branches to shield itself from parasitic wasps during pupation [31]. Moreover, these shelters protect insects from environmental stressors, including high winds, heavy rainfall, and extreme temperatures. By regulating temperature and humidity, shelters provide a stable microenvironment, fostering conditions conducive to insect growth, development, and reproduction [32]. The efficacy of entomopathogenic microorganisms in pest control depends on direct contact with the host insect. However, physical shelters, such as silk webs, can impede microbial invasion by creating a barrier between the insect and potential pathogens. It follows that the presence of such physical barriers, particularly silk webs, may influence the success of microbial pest control, although further investigation is warranted.

*Hyphantria cunea*, a global quarantine pest, has invaded over 30 countries and territories [33]. The formation of a silk web is characteristic of the early larval stages of *H. cunea*. *B. bassiana*, a key biocontrol fungus, is widely used to manage *H. cunea* populations [34]. The hypothesis posited is that “the silk web acts as a physical barrier, reducing the efficacy of *B. bassiana* in controlling *H. cunea* larvae.” To test this hypothesis, the following experiments were conducted: (1) After removal of the silk web, larvae were treated with a *B. bassiana* conidia suspension, and the mortality rate was assessed to determine the effect of the silk web on larval mortality. (2) Scanning electron microscopy (SEM) was used to enumerate *B. bassiana* conidia deposited on the larval cuticle, so as to determine whether the presence of the silk web alters the conidial exposure actually experienced by the larvae. (3) The oxidative stress parameters, nutrient content, expression of energy metabolism-related genes, and innate immune responses of *H. cunea* larvae treated with *B. bassiana* were measured following silk web removal, to explore the mechanisms by which the silk web influenced the lethality of *B. bassiana*. This study aims to elucidate the impact of the silk web barrier on the biocontrol efficiency of *B. bassiana* and provide insights into the role of silk webs in pest outbreaks.

## 2. Materials and Methods

### 2.1. Preparation of Beauveria bassiana Conidia Suspension

*B. bassiana* (bio-21839; CAS: 63428-82-0), purchased from Beijing Baiou Bowei Biotechnology Co., Ltd. (Beijing, China), was cultured on potato dextrose agar (PDA) medium at 25 ± 1 °C, with a relative humidity of 70 ± 5% and a photoperiod of 12:12 (L:D). After 14 d of incubation, conidial suspension was prepared. The conidial concentration was adjusted to 1 × 10^8^ conidia/mL using a hemocytometer and a sterile 0.1% Tween-80 solution. Conidial viability was assessed by plating 0.1 mL of the conidial suspension on PDA and incubating at 25 °C for 24 h; germination was ≥95% before use. According to the results of the previous pre-experiment, the infection mortality rate of *B. bassiana* on the 3rd-instar larvae of *H. cunea* at this concentration is between 50% and 60%, which can effectively distinguish the differences among different treatment groups.

### 2.2. Beauveria bassiana Inoculation

In mid-to-late June during the population outbreak of *H. cunea*, poplar branches bearing intact larval silk webs were collected from the epidemic area in Xintaizi Town, Tieling City, Liaoning Province. After being transported to the laboratory, each branch was vertically inserted into 1.5 L water bottles filled with clean water for temporary rearing. There was one branch per bottle, and each branch carried one independent silk web containing *H. cunea* larvae at the early third instar. Each individual silk web was treated as one independent biological replicate in this study. To ensure the rigor of the subsequent experiments, the sizes of these silk webs were similar, and each contained 90–110 larvae. Due to the above reasons and the high demand for silk webs, these silk webs were collected from the same poplar forest rather than the same tree. The branches were divided into two groups: one group retained the silk web (SW), while the other had the silk web removed (RSW). Each silk web was gently peeled off along its base with sterile forceps to ensure no damage to the larval body surface and the integrity of the twigs. The features of the *H. cunea* silk web are detailed in the Supplementary Material. The schematic diagram of the *H. cunea* silk web is shown in Figure S1. A 5 mL suspension of *B. bassiana* conidia, 0.1% (*v*/*v*) Tween-80, or sterile water was applied to each silk web using a spray bottle, or directly onto the larvae in the RSW group following web removal. During spraying, a fixed distance and consistent pressure were maintained to ensure even coverage of the entire web, and the dosage was consistent across all treatment groups. Six experimental groups were established: Bb + SW (*B. bassiana* + silk web), CK + SW (sterile water + silk web), TW + SW (Tween-80 + silk web), Bb + RSW (*B. bassiana* + silk web removal), CK + RSW (sterile water + silk web removal), and TW + RSW (Tween-80 + silk web removal). After treatment, to ensure that the larvae had sufficient food, 2–3 fresh branches were added to each water bottle. The branches were enclosed in gauze netting. Conidial attachment to the larvae was observed 24 h post-treatment. Larval mortality in all groups was recorded after 6 d. The relative reduction in *B. bassiana*-induced larval mortality associated with the presence of the silk web was calculated as follows: Relative mortality reduction = (1 − larval mortality rate of SW group/larval mortality rate of RSW group) × 100%. This index is derived solely from mortality data and expresses the proportional difference in mortality outcome between web-bearing and web-removed larvae; it carries no implication regarding the physical interception of the sprayed suspension, or of the conidia within it, by the silk web. Ten dead larvae were selected from each of the Bb + SW group and Bb + RSW group. They were placed in a 25 °C incubator for 14 d. It was observed that white mycelia and conidia grew on the surface of all the larvae, thereby confirming that the larval death was caused by the infection of *B. bassiana* (Figure S2). In the SW group, each individual silk web was treated as one independent biological replicate, and the larvae in each silk web fluctuated between 90 and 110. Each repeat in the RSW group consists of 100 larvae (Table S2).

### 2.3. Sample Preparation and Scanning Electron Microscopy in Hyphantria cunea After Inoculation with Beauveria bassiana

Bb + SW and Bb + RSW group *H. cunea* larvae samples treated with *B. bassiana* for 24 h were selected. Samples were immersed in 2.5% glutaraldehyde and fixed at 4 °C for 24 h, then rinsed 3 times with the phosphate buffer (pH 7.2). Dried with a critical point dryer (EM CPD300, Leica). Samples were fixed with conductive adhesive and sputter-coated with a 10–20 nm gold film using an ion sputtering coater (EM SCD005, Leica). Observed at 10 kV acceleration voltage and 8–10 mm working distance using a field emission scanning electron microscope (SU8010, Hitachi, Tokyo, Japan). Conidia count per unit area (units: per/mm^2^) was statistically analyzed with ImageJ1 software by selecting 5 random fields of view per sample and taking the average value. Four independent biological replicates were established for each treatment group, with each individual silk web regarded as one independent biological replicate. Five larvae were randomly sampled from each silk web for observation (Table S2).

### 2.4. Determination of Cuticular Protein Gene Expression in the Larval Body Wall

After a 6-day treatment with *B. bassiana*, surviving *H. cunea* larvae from the Bb + SW, CK + SW, TW + SW, Bb + RSW, CK + RSW, and TW + RSW groups were selected for gene expression analysis. The reason for choosing this time point for parameter measurement is that the mortality rate of the third-instar larvae of *H. cunea* infected with *B. bassiana* was about 50% within 6 d. Measuring the physiological indicators of the larvae at a mortality rate of about 50% would be more representative. In brief, these larvae were placed in pre-labeled RNase-free centrifuge tubes. Subsequently, the centrifuge tubes containing the larvae were quickly placed in liquid nitrogen for rapid freezing. These larvae were taken out of the liquid nitrogen and then placed in a pre-cooled mortar to be quickly ground into a uniform powder-like tissue. Liquid nitrogen was continuously added to prevent RNA degradation caused by the heat generated during the grinding process. Total RNA was extracted from the powder-like tissue using a commercially available RNA extraction kit (Beyotime Biotechnology). RNA quality and concentration were assessed, and the total RNA was then reverse transcribed into complementary DNA (cDNA) using a reverse transcription kit (Takara Bio, Beijing, China). The relative expression levels of target genes were quantified using the KOD SYBR^®^ quantitative polymerase chain reaction (qPCR) kit (Toyobo, China), with cDNA as the template. The RT-qPCR reaction system consisted of 20 µL, including 10 µL of KOD SYBR^®^ qPCR mix (Toyobo, China), 0.6 µL of forward primer, 0.6 µL of reverse primer, 1 µL of cDNA, and 7.8 µL of DEPC-treated water. The expression of *CP2*, *CP3*, *CP1M*, *CP2M*, *LCP30*, and *CPT4P* genes was measured using RPS16 as a reference gene. Gene expression was calculated using the 2^–ΔΔCt^ method. Primer sequences for the target genes are given in Table S1. Four independent biological replicates were used for gene expression quantification in each treatment. Each replicate corresponded to an individual silk web, and five larvae were randomly sampled from each web and pooled into a single testing sample (Table S2).

### 2.5. Determination of Cellular Immunity Level in Hyphantria cunea Larvae

After 6 d of *B. bassiana* treatment, surviving *H. cunea* larvae from the Bb + SW, CK + SW, TW + SW, Bb + RSW, CK + RSW, and TW + RSW groups were selected. The distal tip of the abdominal proleg of each larva was excised with sterile surgical scissors, and the naturally exuded hemolymph was collected with a 2 μL micropipette. The hemolymph obtained from each group was subsequently used to assess hemocyte counts and melanization activity. For hemocyte counting, the hemolymph was diluted 1.5-fold in supersaturated PTU-Ringer’s saline, and 10 μL of the mixture was placed on a counting chamber. The hemocytes were counted under an optical upright phase contrast microscope. Six independent biological replicates were performed for hemocyte observation. For each replicate derived from an individual silk web, hemolymph extracted from five larvae was pooled as one testing sample (Table S2). Melanization activity was evaluated by dropping 2 μL of hemolymph onto a white plastic plate and observing the melanization after 20 min. Melanization was classified into five grades: 0, hemolymph white or light yellow; 1, hemolymph yellow; 2, hemolymph yellow-brown; 3, hemolymph brown; and 4, hemolymph black. Melanization activity = ∑(representative value of melanization grade × number observed in each grade)/total number observed [35]. Each individual larva represented one biological replicate for hemocyte analysis, and 15 replicates were analyzed for each silk web treatment group (Table S2). Encapsulation activity was evaluated using the gel bead injection method described by [35]. Gel bead encapsulation was examined under an optical upright phase contrast microscope, and the thickness (L) of the capsule and the diameter (D) of the gel beads were measured using ImageJ software. The encapsulation of gel beads was classified into 13 grades based on the following criteria: Grade 1 indicated no encystment or only minimal cell attachment; Grade 2 denoted incomplete encapsulation; Grade 3 corresponded to 0 < L/D < 0.1; Grade 4 to 0.1 < L/D < 0.2; and so on, with Grade 13 representing L/D > 1. Encapsulation activity = Σ*n*, where *n* refers to the encapsulation grade. Thirty individual replicates were randomly selected from the four independent biological replicates of both the silk web group and the removed web group, and each replicate consisted of a single gel bead (Table S2). Total RNA extraction of larval whole bodies, cDNA synthesis, and RT-qPCR were conducted as outlined in Section 2.4. The expression of cellular immune regulatory genes was measured, including *Galectin-1*, *Galectin-2*, *Hemolin-1*, *Hemolin-2*, *DSCAM*, and *PPO-2*. qPCR primer sequences for these genes are shown in Table S1. Four independent biological replicates were used for gene expression quantification in each treatment. Each replicate corresponded to an individual silk web, and five larvae were randomly sampled from each web and pooled into a single testing sample (Table S2).

### 2.6. Determination of Humoral Immunity Level in Hyphantria cunea Larvae

Total RNA of larval whole bodies was extracted, cDNA was synthesized, and RT-qPCR assays were performed following the procedure outlined in Section 2.4. The expression of humoral immunity-related genes was assessed, including immune recognition genes from the IMD and Toll pathways (*GNBP1*, *GNBP3-1*, and *PGRP-SA*), signal transduction genes (*Relish*, *Myd88*, *Tube*, *TAK*, *Dorsal*, *IMD*, and *Toll*), and antimicrobial peptide genes (*Lebocin*, *Gloverin*, *Cecropin*, *Lysozym*, and *Attacin*). The RT-qPCR primer sequences for these genes are shown in Table S1. Four independent biological replicates were used for gene expression quantification in each treatment. Each replicate corresponded to an individual silk web, and five larvae were randomly sampled from each web and pooled into a single testing sample (Table S2).

### 2.7. Determination of Antioxidant and Oxidative Damage Parameters in Hyphantria cunea Larvae

The secretion of toxins causing oxidative damage to larvae is the main mechanism by which *B. bassiana* exerts insecticidal activity. This oxidative damage is targeted at the overall tissue of the larvae. In this regard, from the perspective of oxidative damage of the larval whole tissues, we evaluated whether the removal of the silk web would exacerbate the toxic effects of *B. bassiana* on the *H. cunea* larvae. In brief, after 6d of *B. bassiana* treatment, surviving *H. cunea* larvae from the Bb + SW, CK + SW, TW + SW, Bb + RSW, CK + RSW, and TW + RSW groups were selected for analysis. Pre-weighed larvae were homogenized with ice-cold saline at a 1:9 (*w*/*v*) ratio. The homogenates were then centrifuged at 2500 rpm (4 °C) for 10 min, and the supernatant was collected for the determination of antioxidant enzyme activities (catalase, CAT; superoxide dismutase, SOD; peroxidase, POD) and the levels of MDA and H_2_O_2_. Enzyme activities were expressed in units of U/mg protein, while MDA and H_2_O_2_ concentrations were measured in nmol/mL and μmol/mg, respectively. CAT activity was assessed using the ammonium molybdate method according to the kit instructions (Product No. A007-1-1). SOD activity was measured by the hydroxylamine method (Product No. A001-1). POD activity was evaluated by colorimetry according to the kit instructions (Product No. A084-1-1). MDA content was quantified using the TBA method (Product No. A003-1), and H_2_O_2_ levels were determined via spectrophotometry according to the kit protocol (Product No. A064-1-1). In addition, pre-weighed *H. cunea* larvae were homogenized with pre-cooled saline (1:4, *w*/*v*) under ice bath conditions. After centrifugation at 12,000 rpm (4 °C) for 5 min, the supernatant was collected for determination of T-AOC (unit: mmol/mg prot) using the FRAP method, as outlined in the detection kit (Product number: A015-3-1). All kits were purchased from Nanjing Jiancheng Biological Co., Ltd. (China). Total RNA extraction, cDNA synthesis, and RT-qPCR were conducted on *H. cunea* larvae following the procedure described in Section 2.3. The expression levels of antioxidant genes (*SOD1*, *SOD2*, *POD1*, *POD2*, *CAT1*, and *CAT2*) and oxidative stress-related genes (*AIFM3*, *BCL11A*, *Metacaspase-1-like*, *Caspase-6*, *Caspase4-1*, and *Caspase4-2*) were analyzed using *RPS16* as a reference gene. Primer sequences for RT-qPCR are provided in Table S1. Four independent biological replicates were used for gene expression quantification in each treatment. Each replicate corresponded to an individual silk web, and five larvae were randomly sampled from each web and pooled into a single testing sample (Table S2).

### 2.8. Determination of Nutrient Content and Energy Metabolism Gene Expression of Hyphantria cunea Larvae

The supernatant used for enzyme activity assessment (Section 2.7) was used to measure glucose, amino acid, and total protein concentrations (μmol/mg) in *H. cunea* larvae. The total protein detection kit (Product No.: A045-4), glucose detection kit (Product No.: A154-1-1), and total amino acid detection kit (Product No.: A026-1) were purchased from Nanjing Jiancheng Biological Co., Ltd. (China). Each experimental group consisted of four replicates, each containing five larvae. Total RNA extraction, cDNA synthesis, and RT-qPCR analysis were conducted on *H. cunea* larvae as described in Section 2.4. The expression levels of energy metabolism-related genes were assessed, focusing on four key regulatory genes involved in glycolysis (*PK*, *HK1*, *HK2*, and *PFK*) and four crucial genes in the tricarboxylic acid cycle (*IDH1*, *IDH2*, *CS*, and *DGDC*). The corresponding RT-qPCR primer sequences are shown in Table S1. Four independent biological replicates were used for gene expression quantification in each treatment. Each replicate corresponded to an individual silk web, and five larvae were randomly sampled from each web and pooled into a single testing sample (Table S2).

### 2.9. Statistics and Analysis

Data processing and statistical analyses were performed using SPSS 26.0 software (SPSS Inc., Chicago, IL, USA). Results are presented as mean ± standard deviation (mean ± SD). According to the biological functional attributes of the measured indicators, all detected variables were preclassified into seven independent functional groups: (1) cuticular protein genes; (2) cellular immunity parameters and related genes; (3) humoral immunity genes; (4) antioxidant defense parameters and related genes; (5) oxidative damage and apoptosis-related parameters and genes; (6) nutrient contents and glycolysis-related genes; (7) tricarboxylic acid cycle-related genes. Pairwise comparisons of quantitative data between the SW and RSW groups were conducted using independent samples *t*-tests. To control for false-positive bias induced by multiple testing within each functional group, the Benjamini–Hochberg false discovery rate (FDR) correction was applied separately to each group. An FDR-adjusted *p* value < 0.05 was considered to indicate a statistically significant difference. The degree of melanization and encapsulation activity were compared between groups using the Mann–Whitney *U* test. Differences in larval mortality across the six groups were examined using one-way analysis of variance (one-way ANOVA), followed by LSD post hoc tests for pairwise multiple comparisons. Figures were generated using GraphPad Prism 8.0 software and the CNSknowall online platform (https://cnsknowall.com).

## 3. Results

### 3.1. Effect of Silk Web Removal on Beauveria bassiana-Induced Larval Mortality

**Figure 1:**
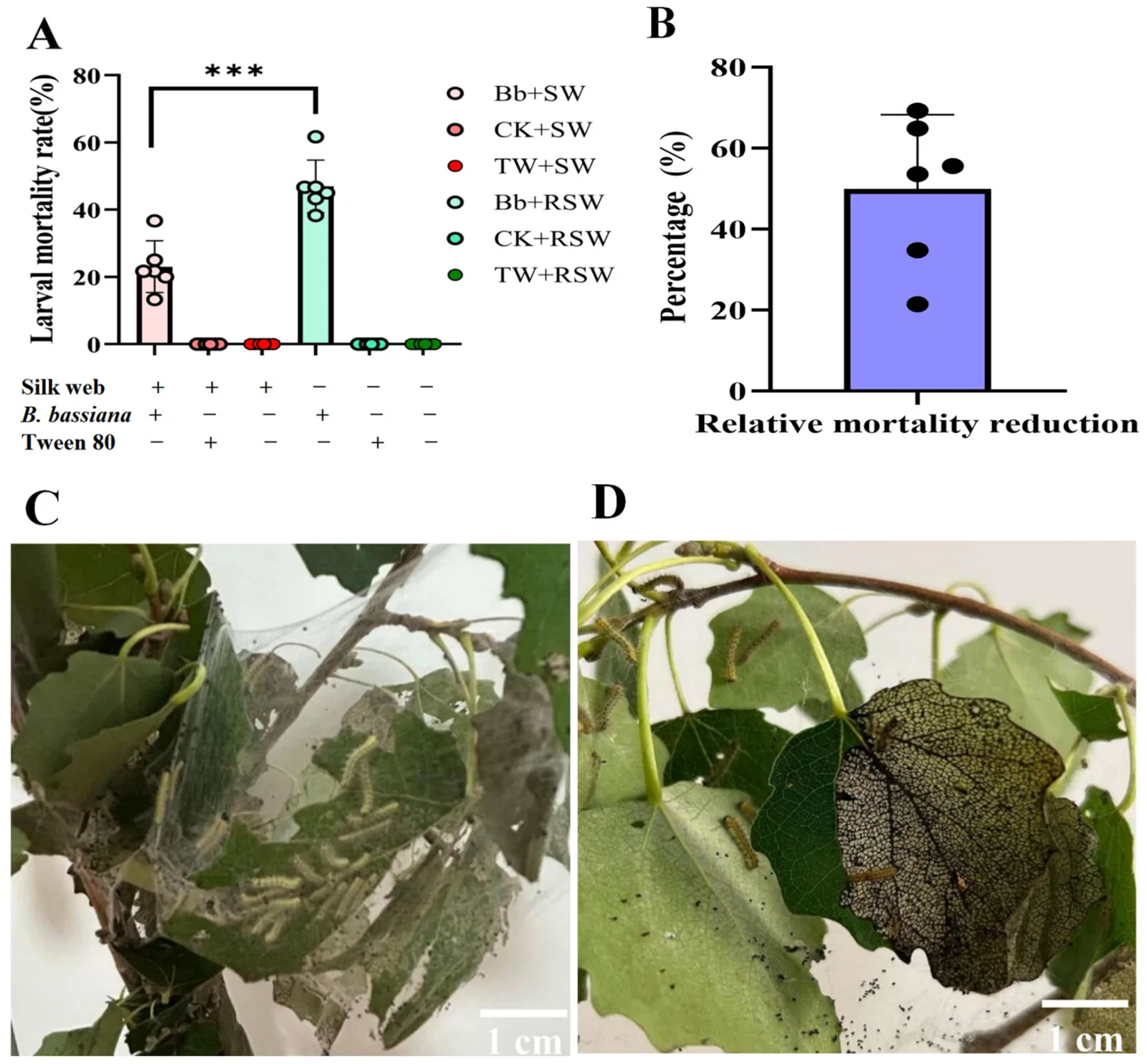
Lethality of *B. bassiana* to *H. cunea* larvae following silk web removal (**A**) and relative mortality reduction by silk web (**B**). The status of *H. cunea* larvae in the Bb + SW (*B. bassiana* + silk web) group (**C**) and Bb + RSW (*B. bassiana* + removal of silk web) group (**D**) after *B. bassiana* treatment. ***, *p* < 0.001.

After a 6-day treatment with *B. bassiana*, the mortality rate of *H. cunea* larvae in the Bb + RSW group reached 46.94 ± 3.20%, more than double that observed in the Bb + SW group (23.06 ± 3.14%) (Figure 1A). The mortality rates of larvae in the CK + SW group, CK + RSW group, TW + SW group, and TW + RSW group were 0% (Figure 1A). Statistical analysis revealed a significant difference in larval mortality between the Bb + RSW group and the Bb + SW group under *B. bassiana* infection, with the Bb + RSW group exhibiting a higher rate compared to the Bb + SW group (*T* = −5.32, *df* = 10, *p* < 0.001). The presence of the silk web corresponded to a 50.88% relative reduction in *B. bassiana*-induced *H. cunea* larval mortality (Figure 1B–D).

### 3.2. Electron Microscope Observation of the Conidia Density on the Larval Body Surface

**Figure 2:**
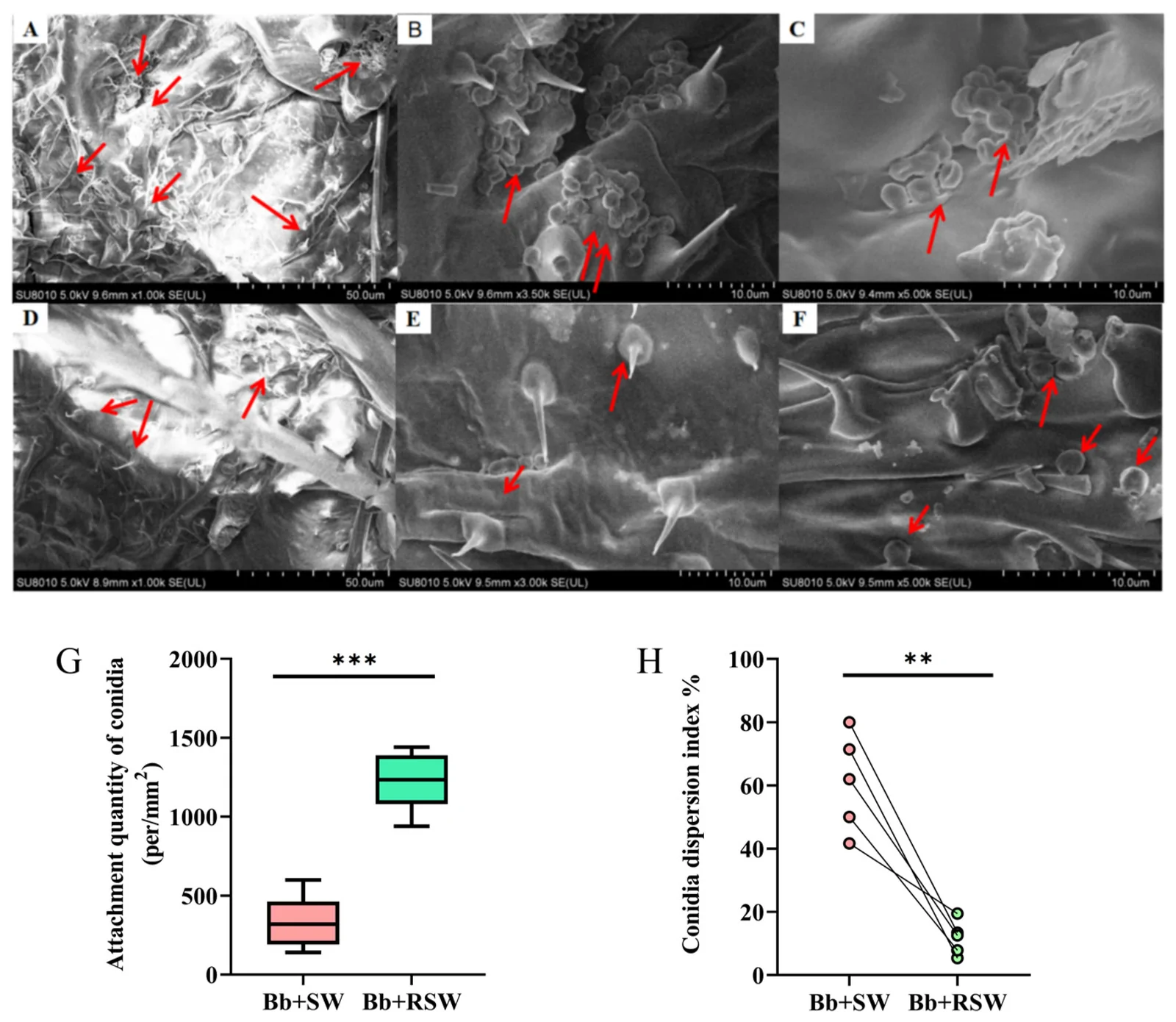
Schematic representation showing the conidia attachment on the larval body surface in the Bb + RSW (*B. bassiana* + removal of silk web) group (**A**–**C**) and Bb + SW (*B. bassiana* + Silk web) group (**D**–**F**). Attachment quantity (**G**) and dispersion index (**H**) of *B. bassiana* conidia on the larval body surface in the Bb + SW and Bb + RSW groups. Red arrows indicate *B. bassiana* conidia. **, *p* < 0.01; ***, *p* < 0.001.

The conidia attachment on the larval body surface in the Bb + RSW group exceeded that in the Bb + SW group. Closer examination revealed that the conidia aggregated within the folds of the *H. cunea* larval body surface in the Bb + RSW group, whereas the conidia were more dispersed and sparser in the Bb + SW group (Figure 2A–F). Compared with the Bb + SW group, the average attachment amount of conidia on the larval surface of the Bb + RSW group increased significantly by 2.8 times (*T* = −3.924, *df* = 6, *p* < 0.01) (Figure 2G). There was a significant difference in the dispersion degree of conidia between the Bb + SW group and the Bb + RSW group (*p* < 0.01). Specifically, the average dispersion index of the Bb + RSW group was 11.56%, which indicated a high degree of aggregation; while the average dispersion index of the Bb + SW group was 60.77%, falling within the range of 30% to 70% indicated local aggregation (Figure 2H).

### 3.3. Response of the Cuticular Defense in Hyphantria cunea Larvae to Beauveria bassiana Infection After Removing Silk Web

**Figure 3:**
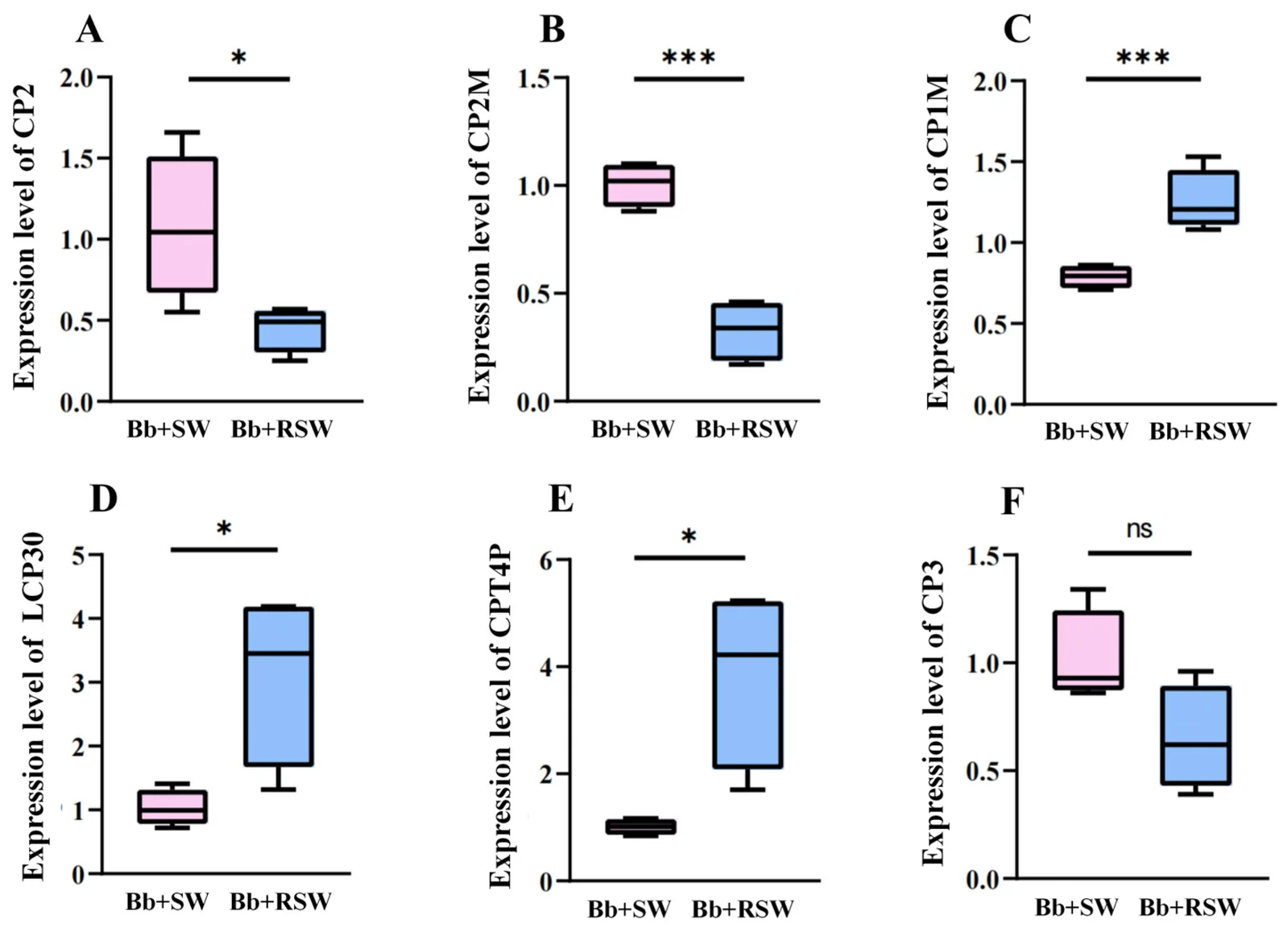
Effect of *B. bassiana* infection on the expression of cuticular protein genes *CP2* (**A**), *CP2M* (**B**), *CP1M* (**C**), *LCP30* (**D**), *CPT4P* (**E**), and *CP3* (**F**) in *H. cunea* larvae after silk web removal. *, FDR-adjusted *p* < 0.05; ***, FDR-adjusted *p* < 0.001; ns, non-significant after FDR correction.

Under *B. bassiana* infection, relative expression levels of cuticular protein genes *CP2* and *CP2M* in the Bb + RSW group were reduced by 58.13% (*T* = 2.626, *df* = 6, *p* = 0.047) and 67.41% (*T* = 7.615, *df* = 6, *p* < 0.001), respectively, compared to the Bb + SW group (Figure 3A,B). In contrast, the expression of *CP1M*, *LCP30*, and *CPT4P* increased by 0.59-fold (*T* = −4.525, *df* = 6, *p* < 0.001), 2.01-fold (*T* = −2.96, *df* = 6, *p* = 0.024), and 2.81-fold (*T* = −3.319, *df* = 6, *p* = 0.024), respectively (Figure 3C–E). No significant difference in *CP3* expression was observed between the Bb + SW and Bb + RSW groups under *B. bassiana* infection (*T* = 2.25, *df* = 6, *p* = 0.065) (Figure 3F). The expression levels of all detected cuticular protein-related genes showed no significant differences between the CK + SW and CK + RSW groups (*p* > 0.05), nor between the TW + SW and TW + RSW groups (*p* > 0.05) (Figures S5I and S6I).

### 3.4. Response of Cellular Immunity Level of Hyphantria cunea Larvae to the Beauveria bassiana Infection After Removing the Silk Web

**Figure 4:**
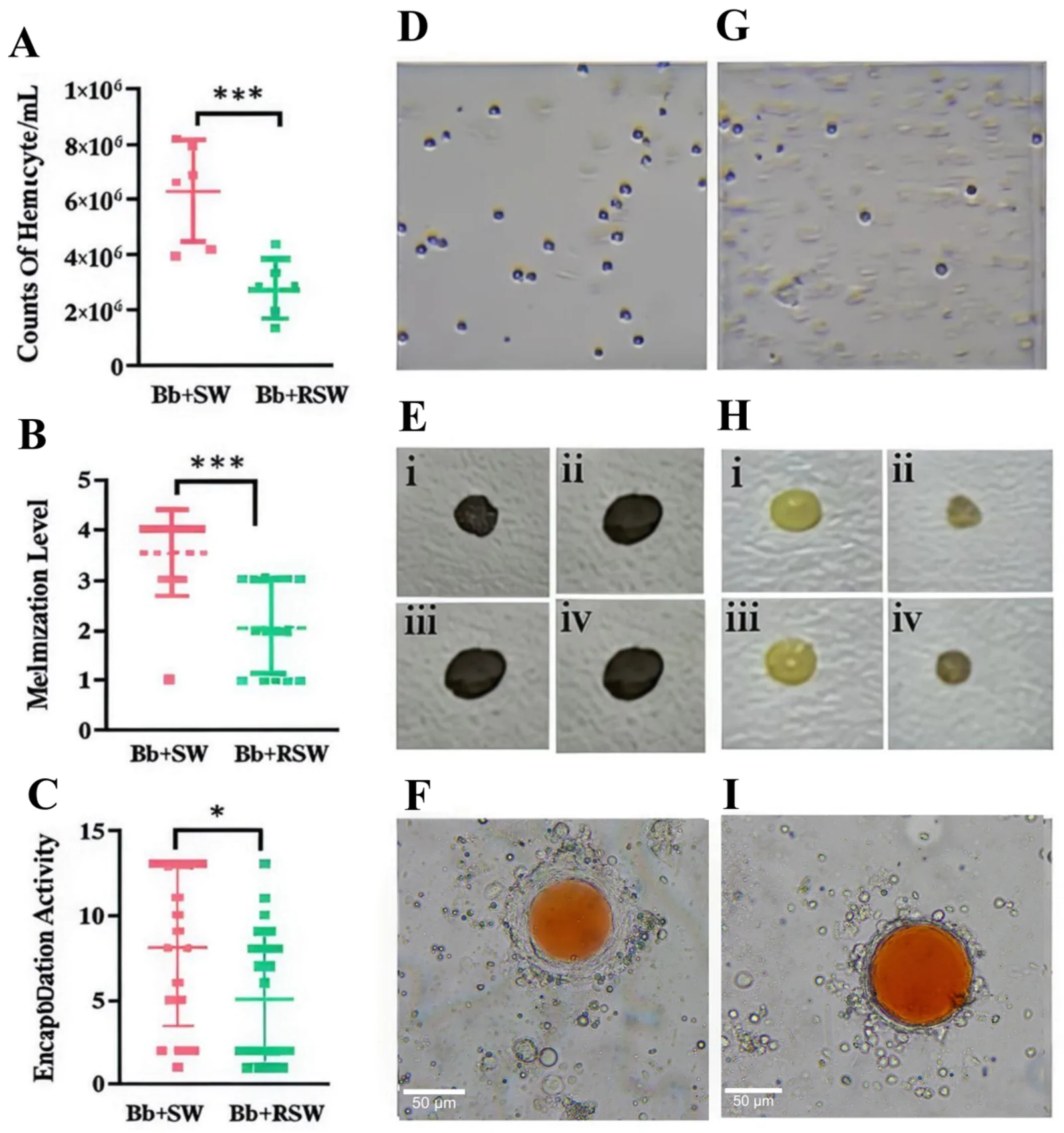
Effect of *B. bassiana* infection on hemocyte count (**A**), melanization (**B**), and encapsulation activity (**C**) in *H. cunea* larvae following silk web removal. Schematic representation of hemocyte count (**D**), melanization (**E**), and encapsulation (**F**) in the Bb + SW (silk web + *B. bassiana*) group. (**E**(**i**)–**E**(**iv**)) represent the four biological replicates for melanization activity determination in the Bb + SW group. (**H**(**i**)–**H**(**iv**)) represent the four biological replicates for melanization activity determination in the Bb + RSW group. Schematic representation of hemocyte count (**G**), melanization (**H**), and encapsulation (**I**) in the Bb + RSW (removal of silk web + *B. bassiana*) group. *, FDR-adjusted *p* < 0.05; ***, FDR-adjusted *p* < 0.001.

**Figure 5:**
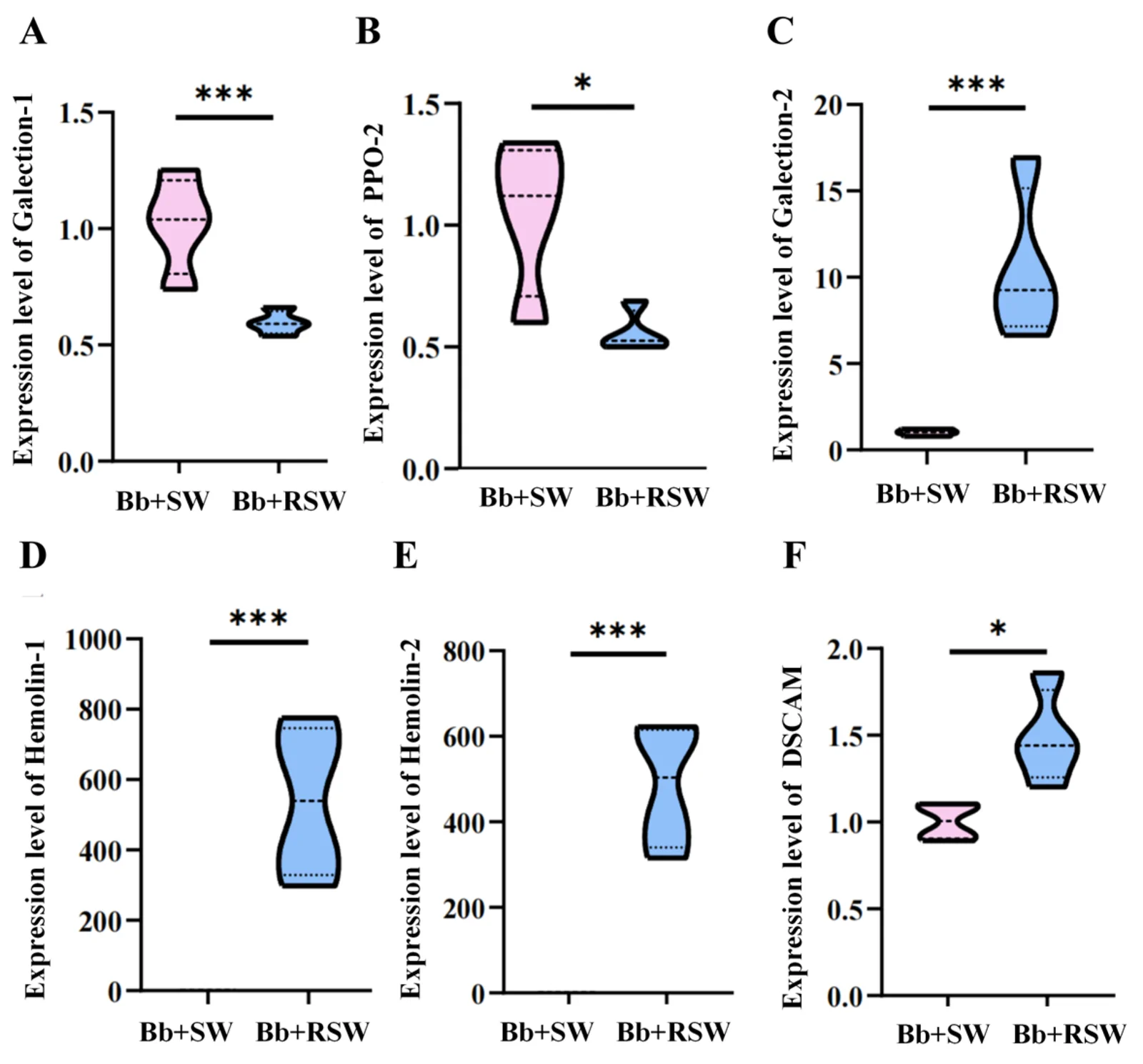
Effect of *B. bassiana* infection on the expression of cellular immunity regulatory genes *Galectin-1* (**A**), *PPO-2* (**B**), *Galectin-2* (**C**), *Hemolin-1* (**D**), *Hemolin-2* (**E**), and *DSCAM* (**F**) in *H. cunea* larvae following silk web removal. The dashed line in the middle indicates the average value. *, FDR-adjusted *p* < 0.05; ***, FDR-adjusted *p* < 0.001.

Under *B. bassiana* infection, the hemocyte count in the visual field of the Bb + SW group was significantly higher than that in the Bb + RSW group, with a 54.75% reduction in hemocytes in the Bb + RSW group compared to the Bb + SW group (*T* = −3.97, *df* = 10, *p* < 0.001) (Figure 4A,D,G). The melanization level of the Bb + RSW group appeared yellowish or brown, while the Bb + SW group exhibited black melanization (Figure 4E,H). Melanization activity in the RSW group was reduced by 40.95% relative to the Bb + SW group (*T* = −4.434, *df* = 27, *p* < 0.001) (Figure 4B). Gel beads were incompletely encapsulated by hemocytes in the Bb + RSW group, whereas in the Bb + SW group, gel beads were largely fully encapsulated (Figure 4F,I). Encapsulation activity in the Bb + RSW group decreased by 38.69% compared to the Bb + SW group (Figure 4C) (*T* = 2.609, *df* = 47, *p* = 0.015). Regarding cellular immune responses, under *B. bassiana* infection, the relative expression of *Galectin-1* and *PPO2* in the Bb + RSW group was down-regulated by 41.52% (*T* = 3.859, *df* = 6, *p* < 0.001) and 46.41% (*T* = 2.901, *df* = 6, *p* = 0.027) (Figure 5A,B), respectively, compared to the Bb + SW group; conversely, *Galectin-2*, *Hemolin-1*, *Hemolin-2*, and *DSCAM* were up-regulated by 9.39-fold (*T* = −4.248, *df* = 6, *p* < 0.001), 522.504-fold (*T* = −4.94, *df* = 6, *p* < 0.001), 463.26-fold (*T* = −6.624, *df* = 6, *p* < 0.001), and 0.48-fold (*T* = −3.289, *df* = 6, *p* = 0.019), respectively (Figure 5C–F). The parameters of all measured cellular immunity showed no significant differences between the CK + SW and CK + RSW groups (*p* > 0.05), nor between the TW + SW and TW + RSW groups (*p* > 0.05) (Figures S3, S4, S5N and S6N).

### 3.5. Response of Humoral Immunity Level of Hyphantria cunea Larvae to the Beauveria bassiana Infection After Removing the Silk Web

**Figure 6:**
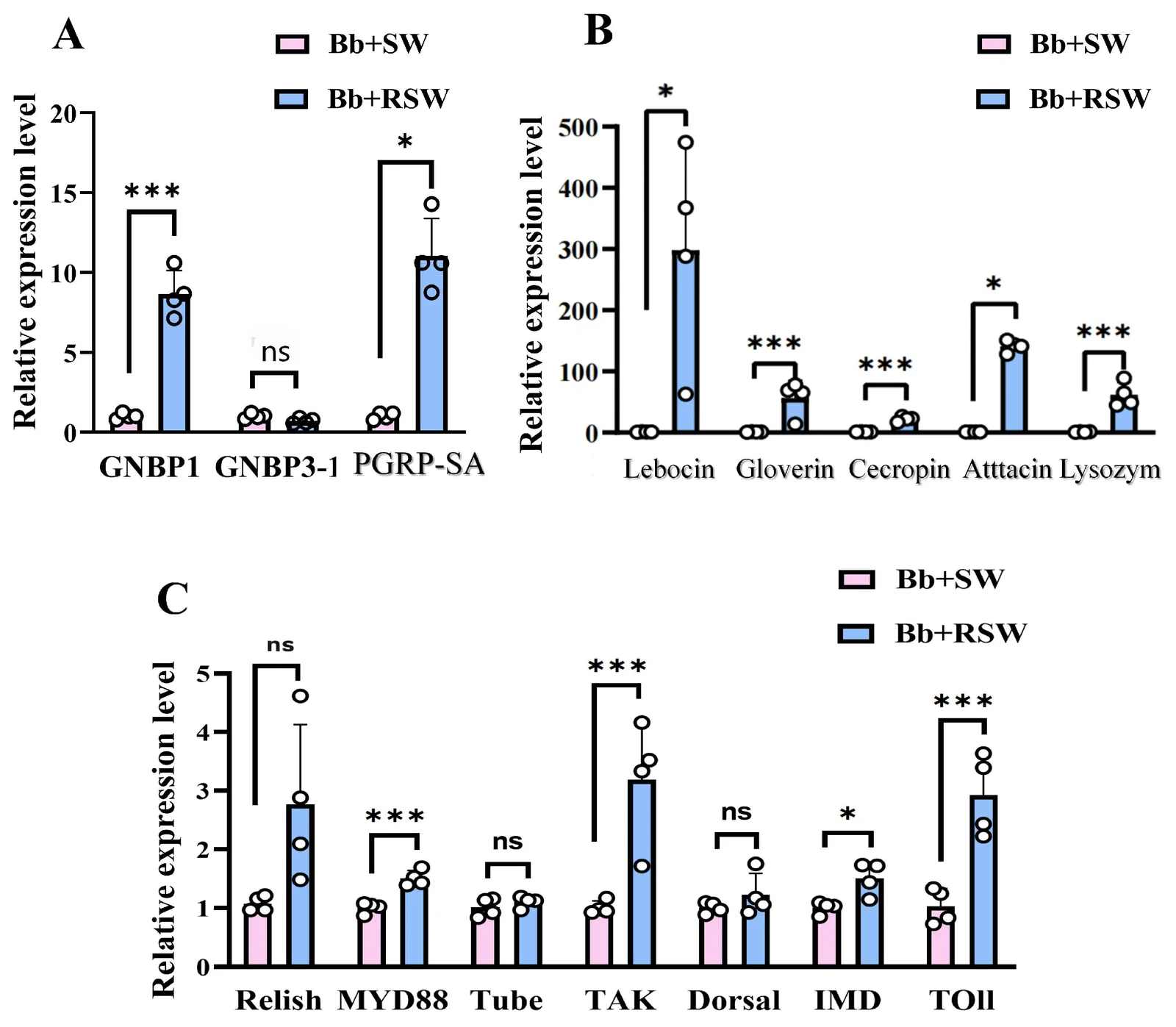
Effect of *B. bassiana* infection on the expression of immune recognition genes (**A**), antimicrobial peptide genes (**B**), and signal transduction genes (**C**) involved in humoral immunity in *H. cunea* larvae following silk web removal. *, FDR-adjusted *p* < 0.05; ***, FDR-adjusted *p* < 0.001; ns, non-significant after FDR correction.

Following *B. bassiana* infection, the expression levels of *GNBP1* and *PGRP-SA* in the Bb + RSW group were upregulated by 7.57-fold (*T* = −7.445, *df* = 6, *p* < 0.001) and 9.88-fold (*T* = −2.942, *df* = 6, *p* = 0.039), respectively, compared to the Bb + SW group. No significant difference in *GNBP3-1* expression was observed between the Bb + SW and Bb + RSW groups under *B. bassiana* infection (*T* = 2.547, *df* = 6, *p* = 0.055) (Figure 6A). In addition, five antimicrobial peptide genes—*Lebocin*, *Gloverin*, *Cecropin*, *Attacin*, and *Lysozym*—were significantly upregulated in the Bb + RSW group under *B. bassiana* infection, with fold increases of 292.21-fold (*T* = −3.404, *df* = 6, *p* = 0.015), 54.42-fold (*T* = −3.829, *df* = 6, *p* < 0.001), 20.88-fold (*T* = −10.613, *df* = 6, *p* < 0.001), 139.92-fold (*T* = −2.951, *df* = 6, *p* = 0.035), and 58.40-fold (*T* = −6.084, *df* = 6, *p* < 0.001), respectively, compared to the Bb + SW group (Figure 6B). Among the seven signal transduction genes examined, expression levels of *Myd88*, *TAK*, *IMD*, and *Toll* in the Bb + RSW group increased by 0.5-fold (*T* = −6.223, *df* = 6, *p* < 0.001), 2.16-fold (*T* = −4.159, *df* = 6, *p* < 0.001), 0.66-fold (*T* = −3.385, *df* = 6, *p* = 0.015), and 1.83-fold (*T* = −4.978, *df* = 6, *p* < 0.001), respectively, in response to *B. bassiana* infection, compared to the Bb + SW group. No significant difference was observed in the expression of *Relish*, *Tube*, and *Dorsal* between the two groups under *B. bassiana* infection (*p* > 0.05) (Figure 6C). The parameters of all measured humoral immunity exhibited no significant differences between the CK + SW and CK + RSW groups (*p* > 0.05), nor between the TW + SW and TW + RSW groups (*p* > 0.05) (Figures S5L,O,Q and S6L,O,Q).

### 3.6. Response of Antioxidant Defense and Oxidative Damage in Hyphantria cunea Larvae to Beauveria bassiana Infection After Removing Silk Web

**Figure 7:**
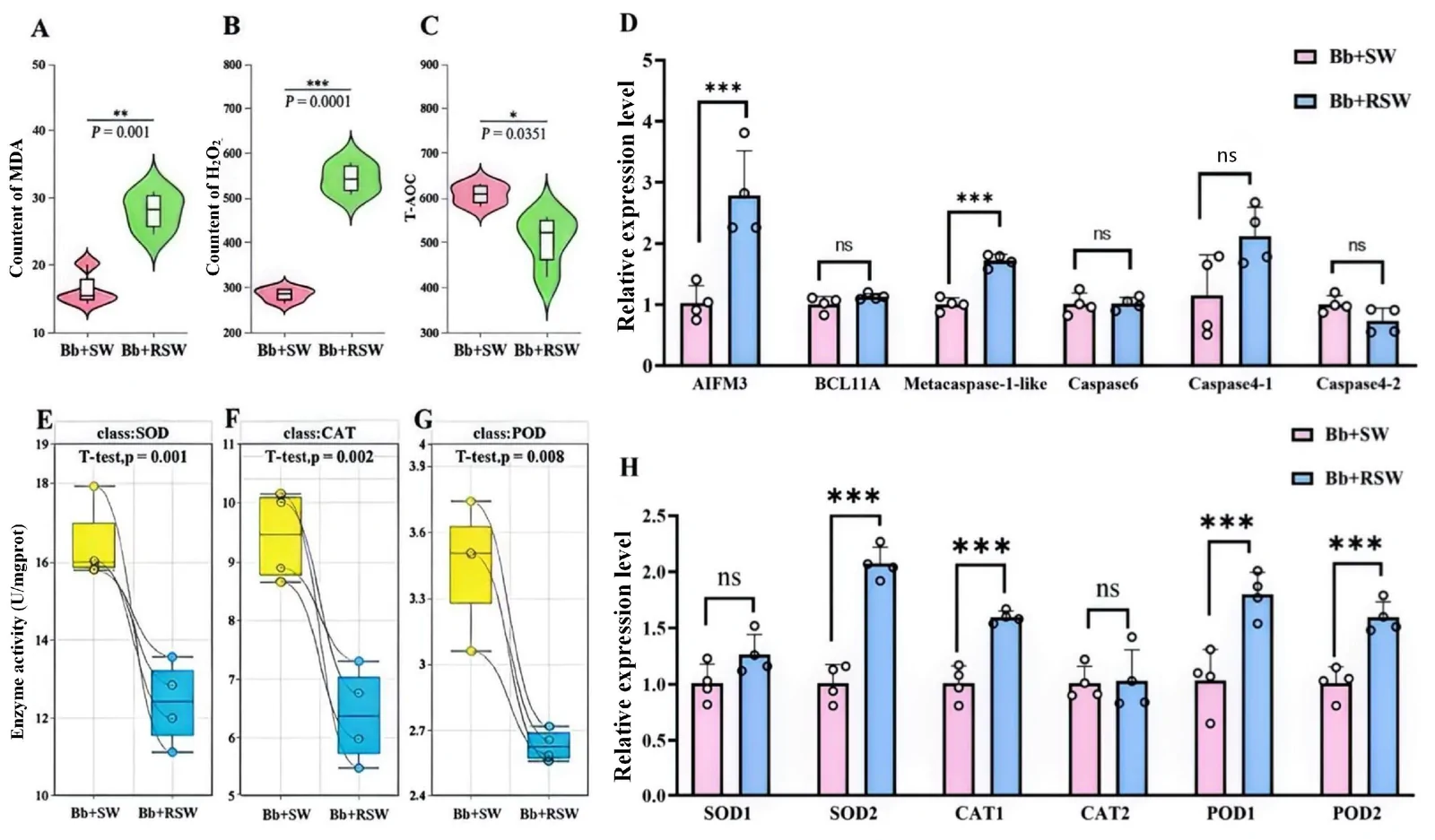
Effect of *B. bassiana* infection on oxidative damage and antioxidant defense in *H. cunea* larvae following silk web removal. (**A**) *MDA*; (**B**) *H*_2_*O*_2_; (**C**) *T-AOC*; (**D**) the expression of apoptosis genes; (**E**) *SOD*; (**F**) *CAT*; (**G**) *POD*; (**H**) the expression of antioxidant enzyme genes. *, FDR-adjusted *p* < 0.05; **, FDR-adjusted *p* < 0.01; ***, FDR-adjusted *p* < 0.001; ns, non-significant after FDR correction.

Under *B. bassiana* infection, the Bb + RSW group exhibited significant decreases in SOD, CAT, and POD enzyme activities by 24.6% (*T* = 5.564, *df* = 6, *p* = 0.001), 32% (*T* = 5.501, *df* = 6, *p* = 0.003), and 23.8% (*T* = 2.554, *df* = 6, *p* = 0.01), respectively, compared to the Bb + SW group (*p* < 0.05) (Figure 7E–G); MDA and H_2_O_2_ levels were notably elevated by 75% (*T* = −4.979, *df* = 6, *p* = 0.002) and 91.4% (*T* = −14.216, *df* = 6, *p* < 0.001) in the Bb + RSW group (Figure 7A,B); the total antioxidant capacity was reduced by 16.79% in the Bb + RSW group (Figure 7C); the expressions of antioxidant genes *SOD2*, *CAT1*, *POD1*, and *POD2* were significantly upregulated by 105.44% (*T* = −9.685, *df* = 6, *p* < 0.001), 58.56% (*T* = −7.183, *df* = 6, *p* < 0.001), 74.09% (*T* = −4.5, *df* = 6, *p* < 0.001), and 57.92% (*T* = −5.848, *df* = 6, *p* < 0.001), respectively. No significant difference in the expression of *SOD1* and *CAT2* was observed between the Bb + RSW and Bb + SW groups under *B. bassiana* infection (*p* > 0.05) (Figure 7H). Among the apoptosis-related genes measured, under *B. bassiana* infection, the expressions of *AIFM3* and *Metacaspase-1-like* in Bb + RSW group were significantly up-regulated by 1.71-fold (*T* = −4.494, *df* = 6, *p* < 0.001) and 0.71-fold (*T* = −9.562, *df* = 6, *p* < 0.001) in the Bb + RSW group, respectively, compared with the Bb + SW group; the expression levels of *BCL11A*, *Caspase6*, *Caspase4-2*, and *Caspase4-1* were not significantly different between the Bb + RSW and Bb + SW groups (*p* > 0.05) (Figure 7D). The parameters of all measured antioxidant defense and oxidative damage showed no significant differences between the CK + SW and CK + RSW groups (*p* > 0.05), as well as between the TW + SW and TW + RSW groups (*p* > 0.05) (Figures S5A,B,E–H,J,P and S6A,B,E–H,J,P).

### 3.7. Response of Nutrient Content and Energy Metabolism in Hyphantria cunea Larvae to the Beauveria bassiana Infection After Removing the Silk Web

**Figure 8:**
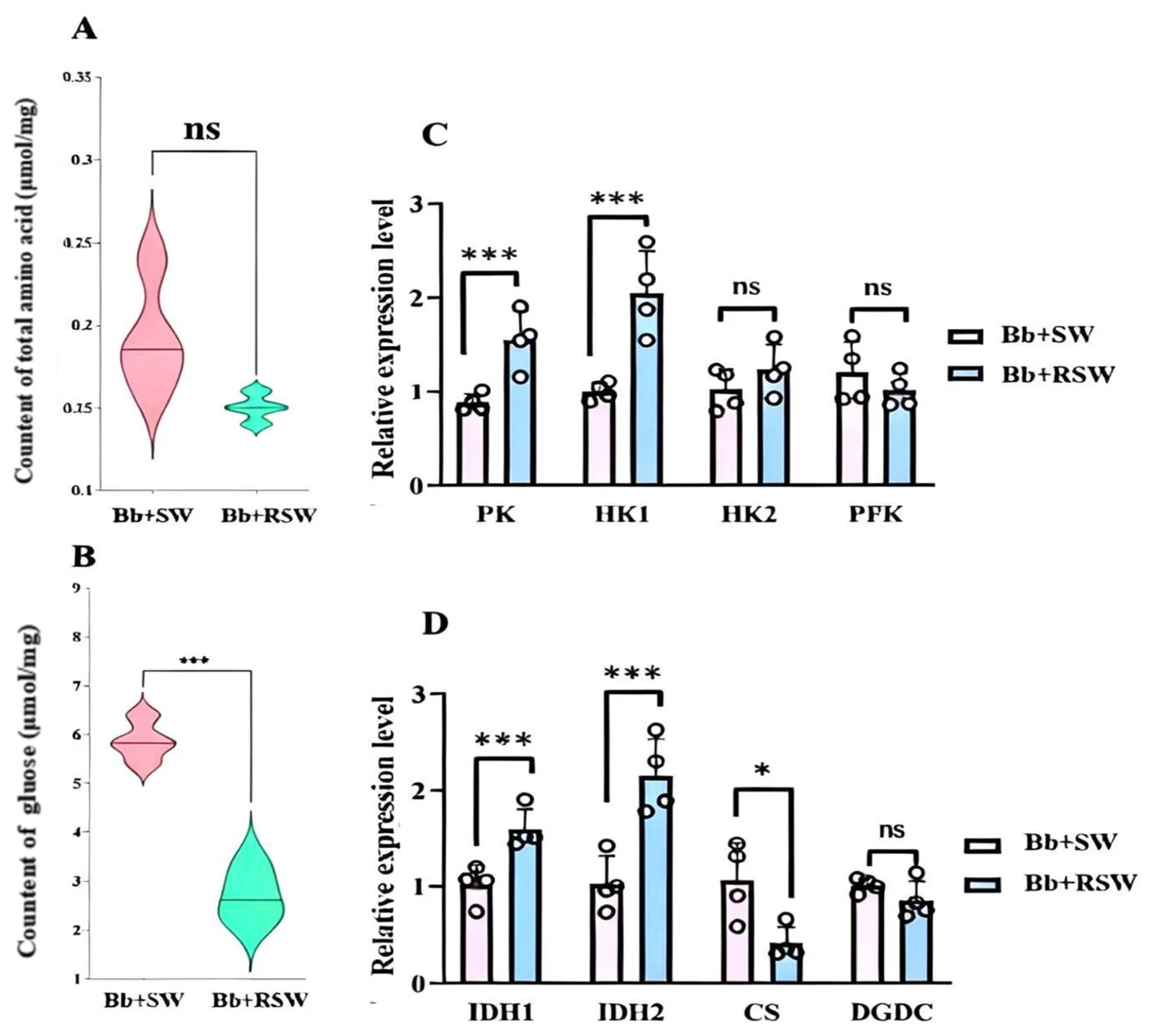
Effect of *B. bassiana* infection on nutrient content and the expression of energy metabolism genes. (**A**) total amino acid content; (**B**) glucose content; key genes in the glycolysis pathway (**C**) and tricarboxylic acid cycle pathway (**D**). *, FDR-adjusted *p* < 0.05; ***, FDR-adjusted *p* < 0.001; ns, non-significant after FDR correction.

Following *B. bassiana* infection, the Bb + RSW group exhibited a significant reduction in glucose and total amino acid levels by 53.99% (*T* = 9.47, *df* = 6, *p* < 0.001) and 21.46% (*T* = 2.447, *df* = 6, *p* = 0.073), respectively, compared to the Bb + SW group (Figure 8A,B). RT-qPCR analysis revealed that, under *B. bassiana* infection, within the glycolysis pathway, the expression of *PK* and *HK1* in the Bb + RSW group increased by 75.35% (*T* = −4.134, *df* = 6, *p* < 0.001) and 104.24% (*T* = −4.565, *df* = 6, *p* < 0.001), respectively, relative to the Bb + SW group (Figure 8C). In the tricarboxylic acid cycle pathway, under *B. bassiana* infection, the expression levels of *IDH1* and *IDH2* in the Bb + RSW group were upregulated by 56.51% (*T* = −4.026, *df* = 6, *p* < 0.001) and 109% (*T* = −4.651, *df* = 6, *p* < 0.001), respectively, compared to the Bb + SW group, while CS gene expression was significantly downregulated by 61.08% (*T* = 3.062, *df* = 6, *p* = 0.029). No significant differences in the expression of *HK2*, *PFK*, and *DGDC* were observed between the Bb + RSW and Bb + SW groups under *B. bassiana* infection (*p* > 0.05) (Figure 8C,D). The parameters of all measured nutrient content and energy metabolism displayed no significant differences between the CK + SW and CK + RSW groups (*p* > 0.05), as well as between the TW + SW and TW + RSW groups (*p* > 0.05) (Figures S5C,D,K,M and S6C,D,K,M).

## 4. Discussion

The silk web serves as a critical physical defense mechanism for insects, protecting them from external stressors [36]. Our findings reveal a significant increase in *H. cunea* larval mortality following *B. bassiana* treatment when the silk web was removed, suggesting that the silk web reduces the efficacy of *B. bassiana* in controlling *H. cunea* larvae. Biological control utilizing entomopathogenic microorganisms is a key strategy for managing *H. cunea* [37]. However, this reduction in efficacy may intensify outbreaks and exacerbate the pest’s impact [38]. It is not difficult to infer that the protective role of the silk web likely contributes to *H. cunea*’s status as a global quarantine pest. Independently, SEM observations revealed a marked increase in the number of *B. bassiana* conidia attached to the body surface of *H. cunea* larvae following silk web removal. These measurements quantify the conidial exposure experienced by individual larvae after the suspension has passed through or around the web; they do not quantify interception by the web itself, and no interception efficiency is inferred from them here. Two main factors may account for the observed difference in conidial exposure: (1) the silk web may physically arrest part of the sprayed conidia, lowering the probability of conidium–larva contact; and (2) the web may impede the dispersal of conidia towards the larvae sheltered within it. The present study does not discriminate between these possibilities. Resolving them will require direct quantification of the conidia retained on the web itself, for example by fluorescent labeling of conidia and measurement of the fluorescence retained on the web, or by wash-off enumeration with recovery correction. This validation remains to be addressed in future work. Effective attachment or colonization of the insect body surface is essential for EPF to exert insecticidal activity [39]. These results are consistent with the observed high mortality of *H. cunea* larvae in the Bb + RSW group and are consistent with the interpretation that the presence of the silk web contributes to the reduced biocontrol efficacy against *H. cunea* larvae. From an applied standpoint, these results suggest that control programs targeting web-forming pests should not rely on conventional spray application alone: mechanical disruption of the web prior to application, formulations with improved penetration of silk structures, or application timed to the pre-web larval stage merit evaluation.

Insects have progressively developed sophisticated defense mechanisms in response to long-term exposure to pathogenic microorganisms [40]. Among these, the body wall defense serves as the primary barrier against pathogenic fungi [41]. Our study demonstrated that, under *B. bassiana* infection, the expression levels of cuticular protein genes *CP1M*, *LCP30*, and *CPT4P* in the Bb + RSW group were significantly higher than those in the Bb + SW group. Cuticular proteins are essential structural components of the insect cuticle. Numerous studies have shown that, when exposed to stressors, insects upregulate the expression of cuticular protein genes to reinforce or stabilize the cuticle, ensuring basic survival. For instance, the expression of cuticular protein genes (e.g., *CP1* and *CP4*) was significantly increased when *Leptinotarsa decemlineata* was exposed to low temperature or drought [42]. A higher expression of the *BmCb10* gene was observed in *Bombyx mori* larvae infected with bacteria [43]. Our results, along with these initial observations, suggest that *H. cunea* larvae in the Bb + RSW group may activate cuticular defenses against *B. bassiana* infection. A plausible explanation for this response is the increased conidial attachment on the larval cuticle in the Bb + RSW group. However, the net effect of these differential expression changes in cuticular protein genes on cuticular defense remains to be further clarified. Notably, compared to the Bb + SW group, the expression levels of *CP2* and *CP2M* under *B. bassiana* infection were significantly reduced in the Bb + RSW group. The ability of *B. bassiana* to breach the host insect’s body wall is fundamental to its insecticidal activity. This suggests that *B. bassiana* may also target and weaken the cuticular defense of *H. cunea* larvae by downregulating the expression of certain cuticular protein genes. The underlying mechanism could involve the secretion of toxins or protein-degrading enzymes, though further investigation is required for confirmation.

Innate immunity, encompassing both cellular and humoral components, serves as the second line of defense for insects against *B. bassiana* infection [44]. In this study, *B. bassiana* infection resulted in a significant reduction in hemocyte count and encapsulation activity in the Bb + RSW group compared to the Bb + SW group, indicating impaired cellular immunity in Bb + RSW larvae. This decline may stem from the silk web removal treatment, which exacerbated *B. bassiana* infection and led to nutrient depletion necessary for hemocyte proliferation. Furthermore, the reduced hemocyte count in the Bb + RSW group directly limited the number of hemocytes available for encapsulation, hindering the encapsulation process. Melanization, a cellular immune response that oxidizes phenolic substances to produce quinones and melanin to eliminate invaders [45], was also significantly impaired in the Bb + RSW larvae upon *B. bassiana* infection. This finding further confirmed the reduced defense capacity of the Bb + RSW group against the pathogen. Conquering the cellular immunity of host insects is the basis for the successful invasion of insect pathogenic microorganisms [46]. For example, after injecting the conidia suspension of *Aspergillus niger*, the number of hemocytes and encapsulation activity in the *Galleria mellonella* larvae decreased significantly [47]. The above findings of this study indicate that removing the silk web is more conducive to the destruction of the cellular immunity of *H. cunea* by *B. bassiana*. Additionally, RT-qPCR analysis revealed a significant decrease in the expression of cellular immunity-related genes *Galectin-1* and *PPO2* in the Bb + RSW group, while *Galectin-2*, *Hemolin-1*, *Hemolin-2*, and *DSCAM* were significantly up-regulated. It is noteworthy that the substantial upregulation of *Hemolin-1* and *Hemolin-2* appears contradictory to the decreased hemocyte count. We speculate that the potential reasons for this apparent discrepancy involve two aspects. On the one hand, this upregulation may represent a compensatory immune response mounted by the larvae to mitigate the immune functional deficit caused by hemocyte reduction; however, this compensatory effect is insufficient to offset the overall attenuation of cellular immunity resulting from the decreased total hemocyte numbers. On the other hand, Hemolin is not synthesized exclusively by hemocytes but can also be expressed and secreted by other immune-related tissues. These changes not only highlight the accelerated *B. bassiana* infection in the Bb + RSW group, which compromises cellular immunity, but also suggest an attempt by the larvae to activate cellular immune responses at the molecular level to counter the rapid infection.

Humoral immunity in insects acts as a defense mechanism against pathogenic microorganisms by synthesizing effectors through the Toll and IMD signaling pathways [48]. The results demonstrated that the expression levels of *GNBP1* and *PGRP-SA* were significantly upregulated in the Bb + RSW group compared to the Bb + SW group after *B. bassiana* infection. As key pattern recognition receptors, *GNBP1* and *PGRP-SA* initiate humoral immune signaling in insects by recognizing pathogen-associated molecular patterns on the surface of invading pathogens. For example, Xu et al. [49] found that after silencing the *βGRP4* gene, the expression levels of the signal transduction genes *IMD* and *Toll* in *B. mori* were significantly decreased, and the sensitivity of *B. mori* to *Beauveria bombysepicus* was significantly enhanced. This suggests that *H. cunea* larvae in the Bb + RSW group can detect *B. bassiana* more efficiently and intensively. Among the seven signal transduction genes analyzed, the expression of *Myd88*, *TAK*, *IMD*, and *Toll* was significantly higher in the Bb + RSW group under *B. bassiana* infection, indicating a stronger activation of the Toll and IMD pathways in response to the fungal threat. Furthermore, the expression of five antimicrobial peptide genes (e.g., *Lebocin*, *Lysozym*) was significantly elevated in the Bb + RSW group. The immune defense function of antimicrobial peptides has been reported in various Lepidoptera insects, such as in *B. mori* larvae responding to *B. mori* nucleopolyhedrovirus, or in *Lymantria dispar* larvae responding to baculovirus and *B. bassiana* [50,51]. These results highlight that the Bb + RSW group exhibits a more robust humoral immune response to *B. bassiana* compared to the Bb + SW group. Combined with pathogen fungal recognition, signal transduction, and effector synthesis, it is not difficult to conclude that *H. cunea* larvae in the Bb + RSW group feel a more intense threat of *B. bassiana* infection and need to mobilize stronger humoral immunity to resist the adverse effects of *B. bassiana*.

Oxidative damage to host insect tissues, induced by toxin secretion, represents a primary mechanism through which EPF exert insecticidal effects [52]. In this study, MDA and H_2_O_2_ levels in the Bb + RSW group were significantly higher than those in the Bb + SW group following *B. bassiana* infection. As key markers of oxidative damage, MDA and H_2_O_2_ directly reflect lipid peroxidation and cellular damage. The results indicated that the absence of silk webs exacerbated the oxidative damage caused by *B. bassiana* in *H. cunea* larvae. Additionally, apoptosis induction is another detrimental outcome of EPF infection in host insects. For example, Ribotoxin secreted by *B. bassiana* induces cell apoptosis in *B. mori* larvae via activating the ROS stress response [53]. RT-qPCR analysis revealed that under *B. bassiana* infection, the expression levels of apoptosis-related genes *AIFM3* and *Metacaspase-1-like* in the Bb + RSW group were significantly higher than those in the Bb + SW group. This molecular evidence supports the notion that removal of the silk web enhances the apoptotic effects of *B. bassiana* on *H. cunea* larvae. These results contribute to the increased sensitivity of *H. cunea* larvae in the Bb + RSW group to *B. bassiana*. In response to oxidative stress or apoptosis, insects have developed an antioxidant defense system primarily based on antioxidant enzymes. However, our results showed that the activities of SOD, CAT, POD, and total antioxidant capacity were significantly lower in the Bb + RSW group compared to the Bb + SW group under *B. bassiana* infection. This suggests that the oxidative damage in the Bb + RSW group exceeded the antioxidant capacity of *H. cunea* larvae. The failure of the antioxidant system also plays a key role in the oxidative damage and apoptosis observed in Bb + RSW group larvae infected with *B. bassiana*. Notably, an elevated expression of antioxidant defense genes in the Bb + RSW group was observed, with significant increases in *SOD2*, *CAT1*, *POD1*, and *POD2*. This indicates that *H. cunea* larvae in the Bb + RSW group attempted to counteract *B. bassiana* infection by activating their antioxidant defense mechanisms. Such an adaptive response likely provides a physiological guarantee for *H. cunea* to resist all aspects of adversity, and is an important reason why *H. cunea* can spread and cause disaster.

Nutritional competition with host insects represents an additional pathogenic mechanism of EPF [17]. Our results revealed that following *B. bassiana* infection, glucose levels in the Bb + RSW group were significantly lower than those in the Bb + SW group. This suggests that the removal of the silk web exacerbates nutrient competition between *B. bassiana* and *H. cunea* larvae. This effect may be attributed to increased colonization and invasion of *H. cunea* larvae by *B. bassiana* conidia, leading to a higher consumption of nutrients and energy by the larvae for defense. The glycolytic pathway and tricarboxylic acid cycle are key routes for maintaining energy and metabolic balance in insects [54]. Compared to the Bb + SW group, expression of the glycolysis regulatory genes *PK* and *HK1* in the Bb + RSW group was significantly upregulated following *B. bassiana* infection. These results suggest that the glycolytic pathway in the Bb + RSW group responds positively to the fungal infestation. A similar pattern was observed in the expression levels of the tricarboxylic acid cycle genes *IDH1* and *IDH2*, further supporting this conclusion. It can be inferred that *H. cunea* larvae in the Bb + RSW group increase energy production by enhancing both glycolysis and the tricarboxylic acid cycle in response to the rapid infection. These results also indicate that removal of the silk web under *B. bassiana* infection exacerbates the disruption of energy metabolism in *H. cunea* larvae. Notably, this study demonstrates a clear correlation between increased conidial exposure and altered physiological responses in larvae after silk web removal, but the direct quantitative dose–response relationship between conidial exposure level and each physiological parameter remains to be further verified in subsequent experiments with gradient conidial concentrations.

## Data Availability

The data underlying this article are available in the article and on request.

## Supplementary Materials

The following supporting information can be downloaded at: https://www.mdpi.com/article/10.3390/insects17101039/s1, Figure S1: A schematic diagram of *H. cunea* silk web; Figure S2: Schematic diagram of hyphal growth phenotype on the cuticle of *H. cunea* larvae killed by *B. bassiana* infection; Figure S3: The level of larval physiological parameters in CK + SW group and CK + RSW; Figure S4: The level of larval physiological parameters in TW + SW group and TW + RSW; Figure S5: The level of larval physiological parameters in CK+SW group and CK+RSW; Figure S6: The level of larval physiological parameters in TW+SW group and TW+RSW; Table S1: Primer sequence of genes; Table S2: Summary of biological and technical replicate designs for all experiments.

## Abbreviations

The following abbreviations are used in this manuscript:

EPF	Entomopathogenic Fungi
Bb	*Beauveria bassiana*
RSW	Silk Web Removal
SW	Silk Web
*H. cunea*	*Hyphantria cunea*
