# Supplementary material for: Silk Web Removal Enhances the Efficacy of Beauveria bassiana Treatment Against Hyphantria cunea: Insights from Immune and Physiological Parameters
Source: Insects. 2026 Oct 10;17(10):1039. doi: 10.3390/insects17101039 (PMC13705550; doi:10.3390/insects17101039)
Supplement: Supplementary file 1 [file insects-17-01039-s001.zip › insects-4533473-supplementary.pdf]

Morphological characteristics of *H. cunea* silk web

The silk web of *H. cunea* larvae is concentrated in the tree canopy area, presenting an irregular mesh or curtain shape. The silk web is thin and light in texture, initially white or light white. It presents a multi-layered fine structure, and has good elasticity and toughness. The size of silk web is related to the instar and quantity of the *H. cunea* larvae. The silk web formed by *H. cunea* larvae at the third instar is the largest and can cover multiple leaves. Silk web where the larvae gather is thick, while the edge is relatively thin.

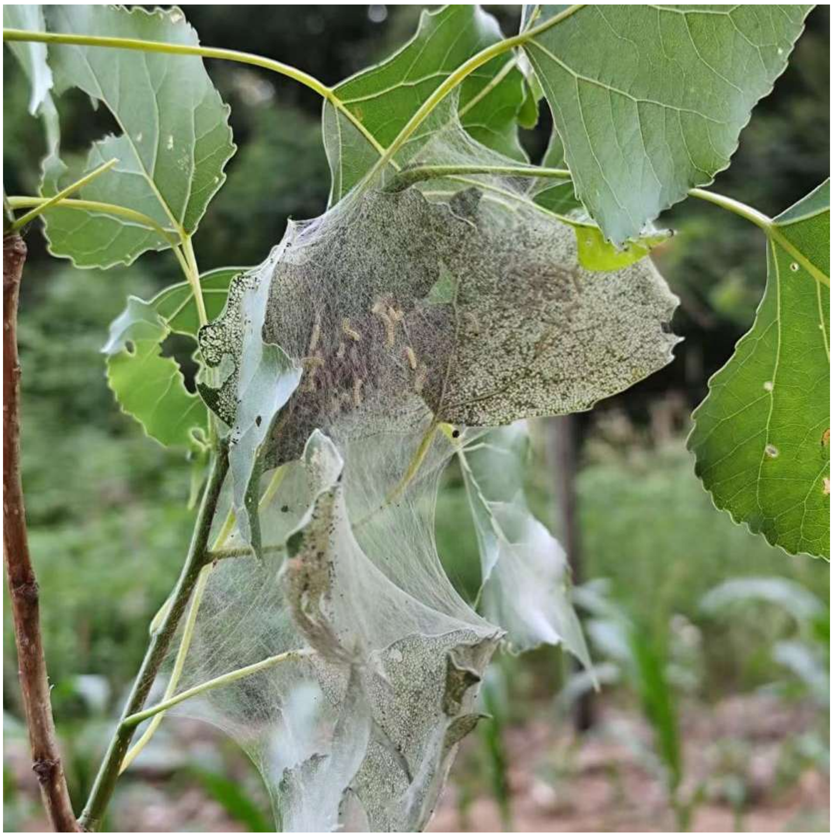

Figure S1 A schematic diagram of *H. cunea* silk web

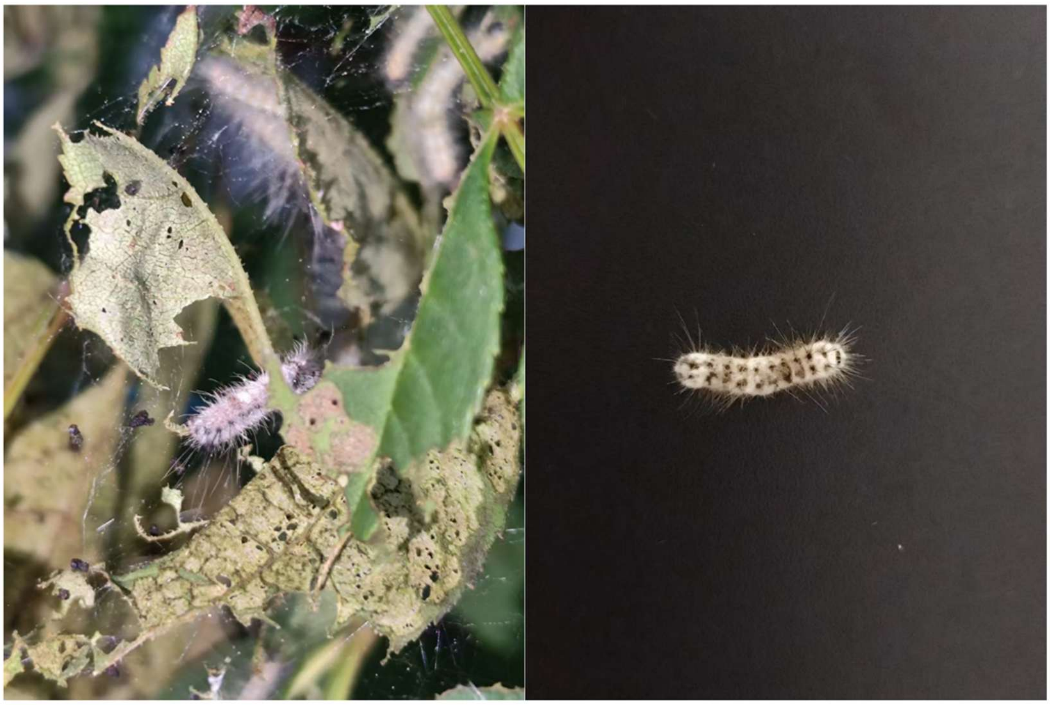

Figure S2 Schematic diagram of hyphal growth phenotype on the cuticle of *H. cunea* larvae killed by *B. bassiana* infection

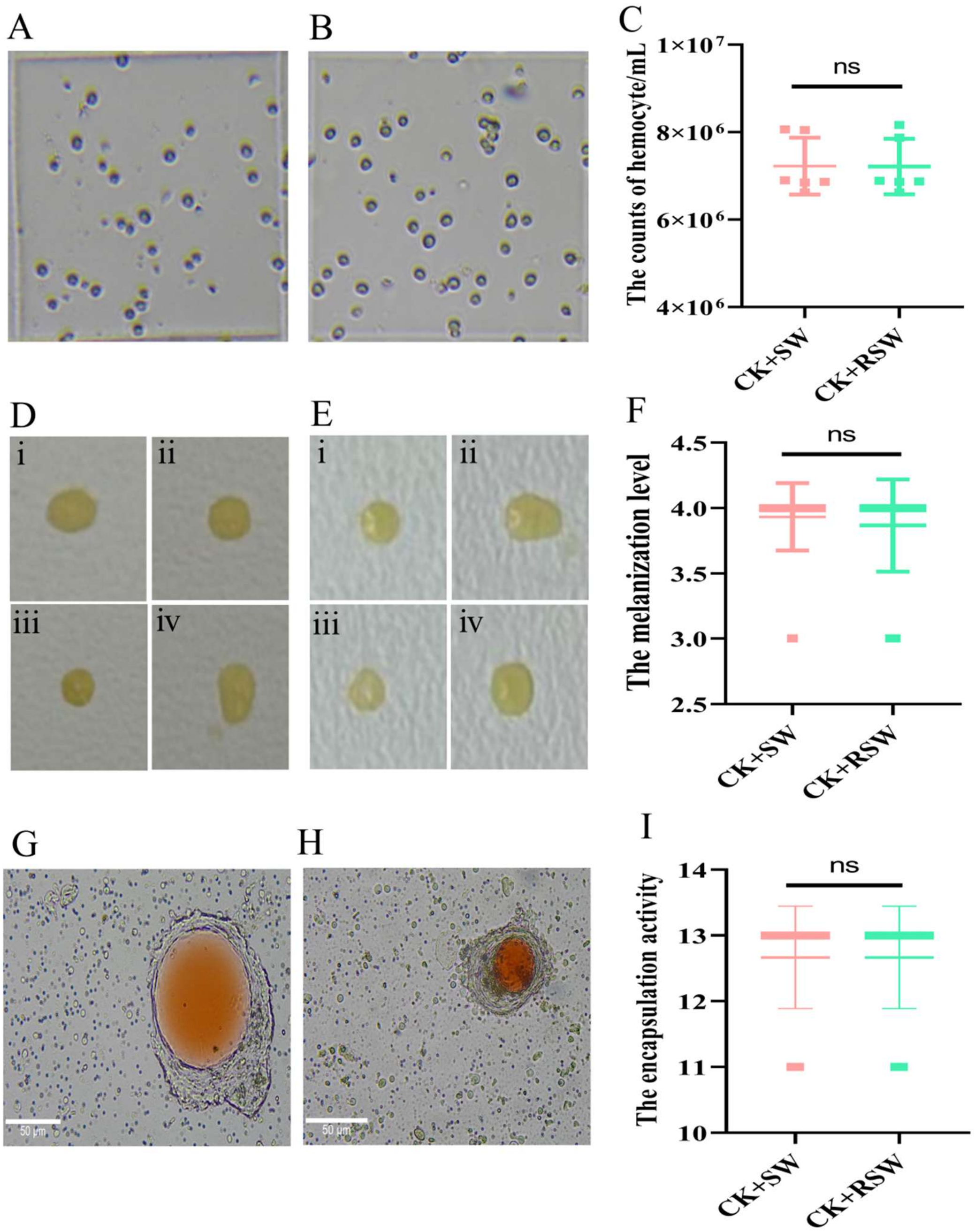

Figure S3 The level of larval hemocyte count (C), melanization (F), and encapsulation activity (I) in the CK+SW group and CK+RSW group. $D_{i\text{-}iv}$ represent the four biological replicates for melanization activity determination in the CK+SW group. $E_{i\text{-}iv}$ represent the four biological replicates for melanization activity determination in the CK+RSW group. Schematic representation of hemocyte count (A and B), melanization (D and E), and encapsulation (G and H) in the CK+SW group and CK+RSW group. ns, non-significant.

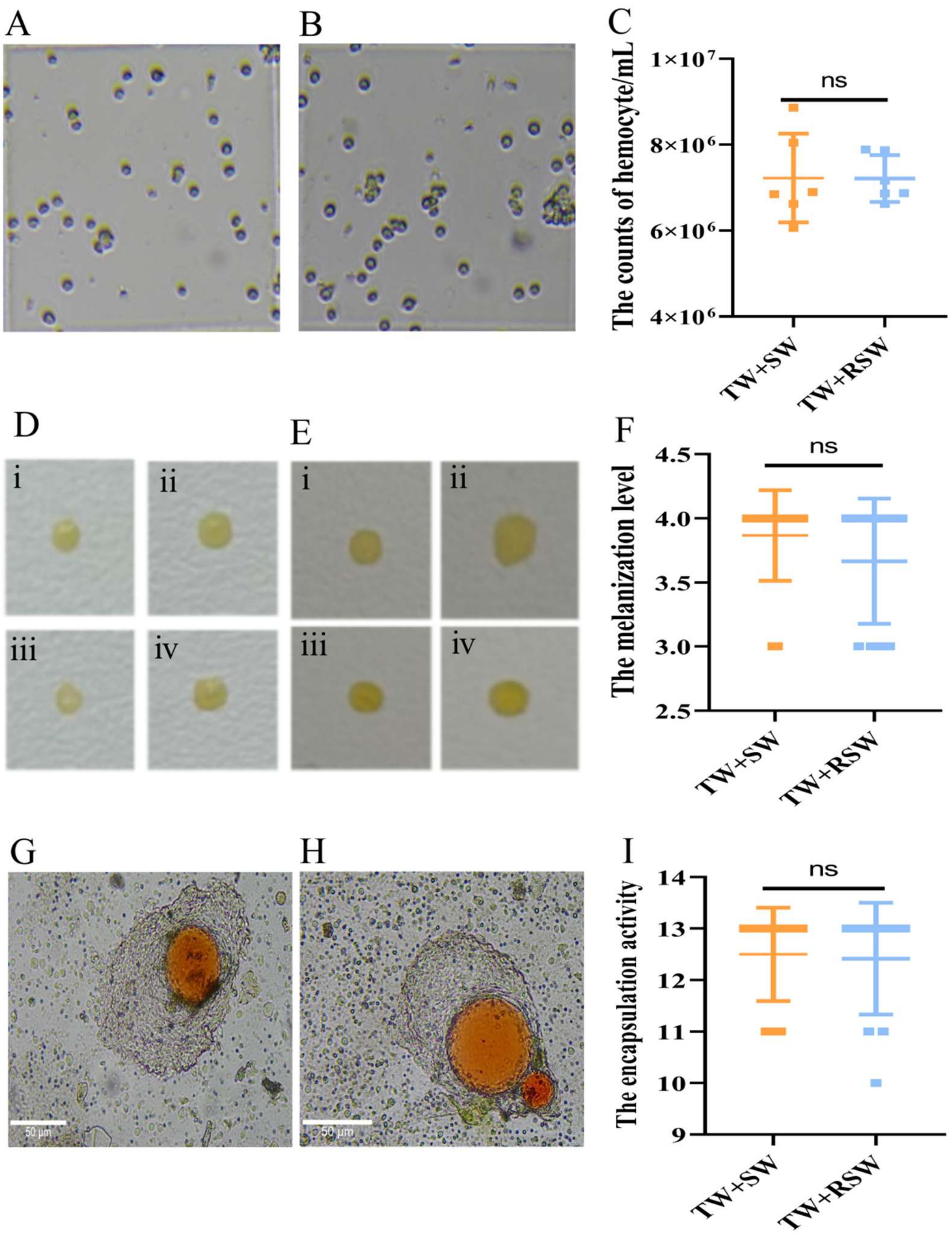

Figure S4 The level of larval hemocyte count (C), melanization (F), and encapsulation activity (I) in the TW+SW group and TW+RSW group. $D_{i\text{-}iv}$ represent the four biological replicates for melanization activity determination in the TW+SW group. $E_{i\text{-}iv}$ represent the four biological replicates for melanization activity determination in the TW+RSW group. Schematic representation of hemocyte count (A and B), melanization (D and E), and encapsulation (G and H) in the TW+SW group and TW+RSW group. ns, non-significant.

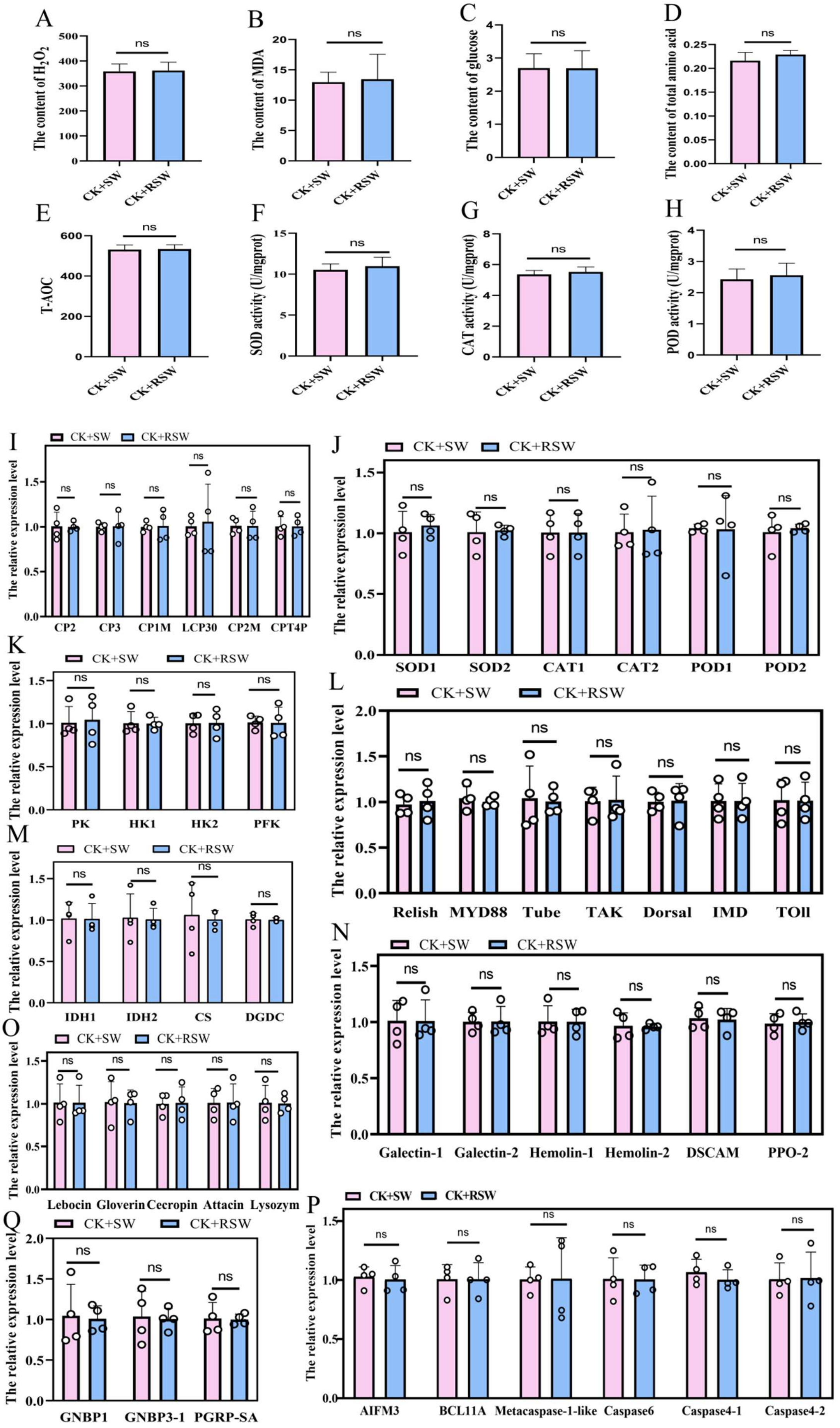
A
The content of H_2O_2
B
The content of MDA
C
The content of glucose
D
The content of total amino acid
E
T-AOC
F
SOD activity (U/mgprot)
G
CAT activity (U/mgprot)
H
POD activity (U/mgprot)
ns
CK+SW
CK+RSW
I
CP2 CP3 CP1M LCP30 CP2M CPT4P
J
SOD1 SOD2 CAT1 CAT2 POD1 POD2
K
PK HK1 HK2 PFK
L
Relish MYD88 Tube TAK Dorsal IMD TOll
M
IDH1 IDH2 CS DGDC
N
Galectin-1 Galectin-2 Hemolin-1 Hemolin-2 DSCAM PPO-2
O
Lebocin Gloverin Cecropin Attacin Lysozym
P
AIFM3 BCL11A Metacaspase-1-like Caspase6 Caspase4-1 Caspase4-2
Q
GNBP1 GNBP3-1 PGRP-SA
The relative expression level

Figure S5 The level of larval physiological parameters in CK+SW group and CK+RSW. (A) H_2O_2, (B) MDA; (C) Glucose content, (D) total amino acid content; (E) T-AOC, (F) SOD, (G) CAT, (H) POD; (I) cuticular protein genes expression levels; (J) antioxidant enzyme genes expression levels; (P) apoptosis-related genes expression levels; (K) glycolysis pathway and (M) tricarboxylic acid cycle pathway key genes expression levels; (L) signal transduction genes, (O) antimicrobial peptide genes, and (Q) immune recognition genes expression levels involved in humoral immunity; (N) cellular immunity regulatory genes expression levels. ns, non-significant differences. ns, non-significant.

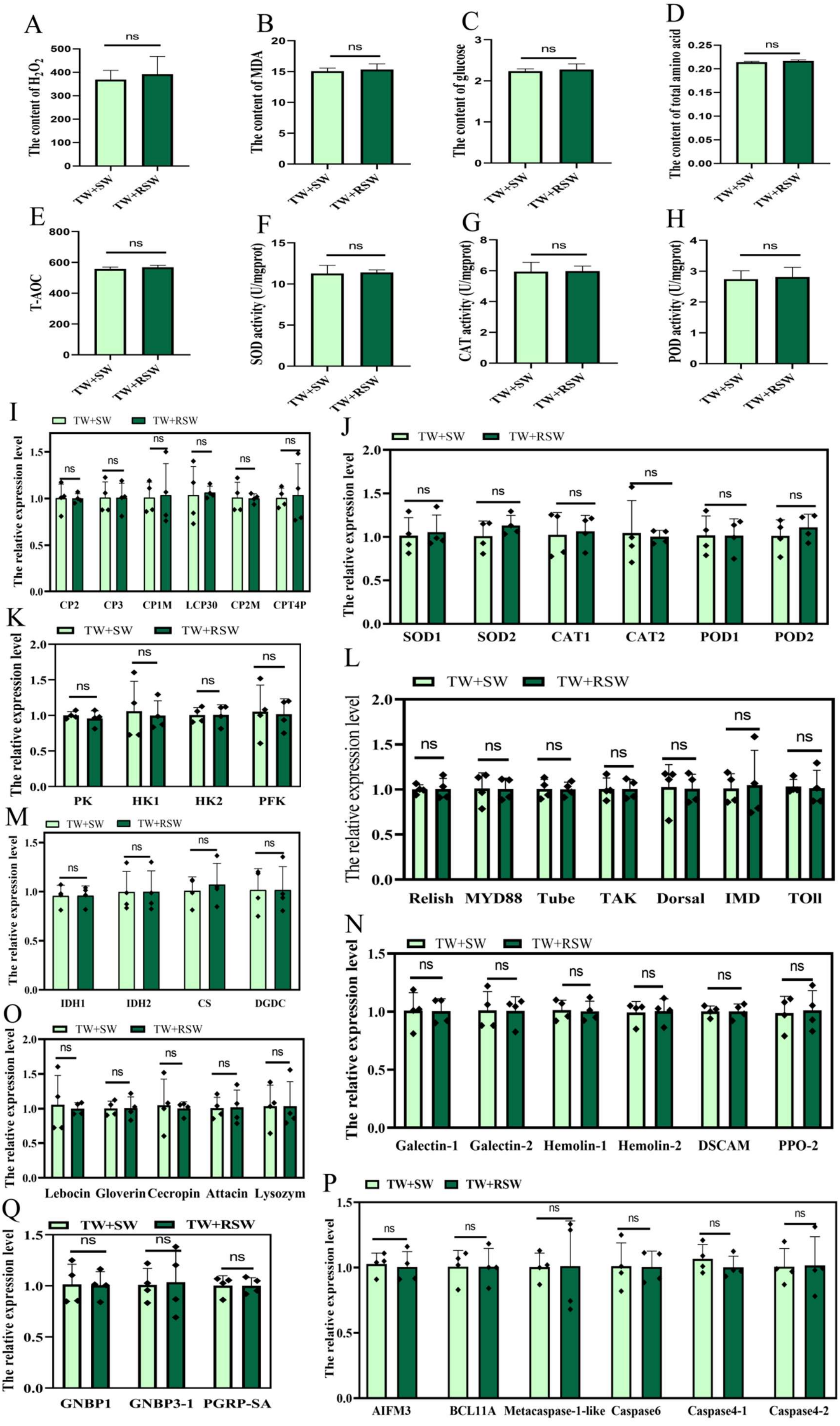

A
The content of H_2O_2
ns
TW+SW
TW+RSW
B
The content of MDA
C
The content of glucose
D
The content of total amino acid
E
T-AOC
F
SOD activity (U/mgprot)
G
CAT activity (U/mgprot)
H
POD activity (U/mgprot)
I
The relative expression level
CP2 CP3 CP1M LCP30 CP2M CPT4P
J
SOD1 SOD2 CAT1 CAT2 POD1 POD2
K
PK HK1 HK2 PFK
L
Relish MYD88 Tube TAK Dorsal IMD TOll
M
IDH1 IDH2 CS DGDC
N
Galectin-1 Galectin-2 Hemolin-1 Hemolin-2 DSCAM PPO-2
O
Lebocin Gloverin Cecropin Attacin Lysozym
P
AIFM3 BCL11A Metacaspase-1-like Caspase6 Caspase4-1 Caspase4-2
Q
GNBP1 GNBP3-1 PGRP-SA

Figure S6 The level of larval physiological parameters in TW+SW group and TW+RSW. (A) H_2O_2, (B) MDA; (C) Glucose content, (D) total amino acid content; (E) T-AOC, (F) SOD, (G) CAT, (H) POD; (I) cuticular protein genes expression levels; (J) antioxidant enzyme genes expression levels; (P) apoptosis-related genes expression levels; (K) glycolysis pathway and (M) tricarboxylic acid cycle pathway key genes expression levels; (L) signal transduction genes, (O) antimicrobial peptide genes, and (Q) immune recognition genes expression levels involved in humoral immunity; (N) cellular immunity regulatory genes expression levels. ns, non-significant differences. ns, non-significant.

Table S1 Primer sequence of genes

Gene name	Forward primer (5′-3′)	Reverse primer (5′-3′)
CP2	TTGCTTATGAGACTGAGAACGGTATAG	GCCATCATCACCAGTGTAGGAATATC
CP3	GCTGCTGGTATCGTCGATGATG	TGTGCTCTAGCGTTGTACTGTTG
CP1M	ATTCCATCCAGTCGGCTCTCAC	CACTTACACAGCCGATGAGAACG
CP2M	GTTGACTACACCGCCGATGATG	GCAGCAATAACCGCAGGAGAAG
LCP30	CGAGAACAAGATCCGTGCTGAAG	CGATGTACTCATAGAAGCCCTTAGATG
CPT4P	ACAAGACACAGAAACAAGAGGGATAC	TCTGAGGATGGTGCCGATGC
AIFM3	AAGCCTGCAAGCACACTGATAGAC	CTCTGCGTACTGAGCAACCACTG
BCL11A	GTCGCACCTTCGCCGTCAC	CCAGGTAGAGCAGGCTGTTTCAC
Caspase4-1	CTGAAGCTGCGGAGCCTTATAGTC	CCTCTGTGATGGGCTGCTCTTATG
Caspase6	AGGAATTGCACGACAACCCATACG	TCGTAGATGTGCTTCGGTCTTTCG
Caspase4-2	GAAGCCGTTGAGCACAAGGATTTC	TGTTGGAGCCATGCGTAAGAACTG
Metacaspase-1-like	TTGAGCCACCTTTGAACAGAGCAG	ATCGTTCGGCGGTGAAGAAATCC
SOD1	AACCCTTATGGGAAACAACACG	CCAGTCCTCTACCGATGATGCT
SOD2	CAGACCCTGTCACCTAAAAGCA	TGTATCCAAGCCAACCCCA
CAT1	GATAGGGAACGCATACCTGAGC	CCTCACAGCCATTGGAGTTCTT
CAT2	GGCGAAAACCACTTCATACG	TCACCGTTACCAATGGCTGT
POD1	Forward: CCTGAGTGGGCAAATGAACC	GCCATAGGTTTCACATCAGCC
POD2	Forward: TTGAAACTATCCCCGAAGGTC	TGGTGTCTTCAGTTTAGCAGGA
Galectin-1	GTCGTCCCATTCCATACCG	TTTCCACTCTGCCACCTCC
Galectin-2	GCGTTTCCGTCAAAGAGTTG	AGCATCAGCCCACATAGCC
Hemolin-1	GTCCTAGCGGCGTGTATAGTTTT	GGAATGCGGTGGAGTAGTTATC
Hemolin-2	Forward:TAACTACTCCACCGCATTCCTA	Reverse:TCCAGCAGCTTTCCAGTCG
DSCAM	Forward:CCCCGACCCCTGACATTAT	Reverse:TGCCAACTGAGTTGCGTGC
PPO-2	CCGAAGTTTGATGAGCGTAACT	CTCCACTTTGAGGCGATAGGTC
PGRP-SA	GCATTCTTGCTTGCGTGTTC	CGTCTGTTTCGGGTTCCTG
GNBP1	CAAGCGTTGAAGCCCAGAG	CGCCTGAAATCGTGCCTAT

GNBP3-1	CAACTGGAACCGCTCAACG	GTTGACCTTGGCGAGTTGC
Relish	GCTAGTTACAGTGTACGGGATG	ATAGAGCCTGGGTTACTTCG
MYD88	TGGCATAGAAACCGAGCAA	GTGACTGAGCCGTGGACAT
Tube	TGCCTGCTTCGTCGCTTGGTTG	TCCATTGCCAGGAAGAACCAAC
Dorsal	CTTGCTGAAGGCGGCTGTA	ACGCATCTTTGTTCTCCATGAG
TAK	AACATGGGCGTTTGTAGGCT	ATGGGAGTTGTTGGTGATAGACAT
IMD	CACATTCTTTAGATCACTTGGCTTCT	CTTTGACGGTGCTCCTTTCG
Toll	ATCTCGGTCCAGTTAATGCC	CGTTCTCCAAAGGACGGTAG
Lysozym	GCGGCAGAAAGACAGACAAG	TCCAACAACAAATCGGCAC
Attacin	AATGGTCACGGTGTATCGG	TGGGCATATTCCTTGAGACA
Cecropin	TCGTGTTCGCTTGTTTCGTC	ACCCAGAACTTGAATAGCAGGA
Gloverin	GGTTTGGCTCACAGTCTTGG	AAAATGTTCTGTTTGTAGCCTCCT
Lebocin	GAGACATCATGCCAACGGTTTA	AAGCCACTGAGGTTCAACATTAGAT
PFK	GGGCGGTTTCTGTGGCTAT	TTGCTGTAAGTCCTTGATCGAG
PK	GCGACGCAAATGTTGGAA	GCATCACGCAGTCAGCACC
HK1	TTTACCTTCTCCTTCCCGATGA	AGACCCCTATCTCTGAGGCATT
HK2	ACCAGAACTCCATCAACCCAG	CCGATTCAATTTCCGATACATA
IDH1	TGATGTTTGGCGTGTCTCGT	CAGTGCCGCAGTGCTAAGAG
IDH2	GGCTTACGGCTCCAGGGTT	CGGTCACTGTCATTCCACTACG
CS	TGTTTGGCGTGTCTCGTGC	TATCAGTGCCGCAGTGCTAAGA
DGDC	GTCGTCTAAATGTCTTGGCTAATG	GGTTCAAACGCTCAATGTAGGT
RPS16	GTGGTCATGTTGCACAGGTT	CTGGACCACCGAACTTCTTG

Table S2 Summary of biological and technical replicate designs for all experiments

**Assay category**	**Specific Measured Indices**	**Number of biological replicates per group (n)**	**Definition of one biological replicate**	**Technical replicate setup**
Phenotypic and infection efficiency assays	Larval mortality	6	Each replicate corresponds to one independently inoculated and reared larval silk web. For the SW group, each web contains 90–110 third-instar larvae; for the RSW group, each independent replicate consists of 100 larvae.	None
Phenotypic and infection efficiency assays	Conidial attachment quantity and dispersion index	4	One independently treated silk web; five larvae were randomly selected from each web for scanning electron microscopy observation.	Five random fields of view per sample were counted, and the mean value was used for analysis.
Gene expression assay (RT-qPCR)	Cuticular protein genes, cellular immunity genes, humoral immunity genes, antioxidant genes, apoptosis-related genes, energy metabolism genes	4	One independently treated silk web. Five larvae were randomly selected and homogenized for total RNA extraction to prepare one cDNA sample for detection.	None

Physiological and biochemical assays	Antioxidant enzyme activities (SOD, CAT, POD, T-AOC), oxidative damage markers (MDA, H_2O_2), nutrient contents (glucose, total amino acids)	4	One independently treated silk web. Five surviving larvae were randomly selected and homogenized, and the supernatant was prepared as one testing sample.	Two parallel measurements were performed for each homogenized sample.
Cellular immunity assays	Hemocyte count	4	One independently treated silk web. Hemolymph collected from five randomly selected larvae was pooled into one testing sample.	None
Cellular immunity assays	Melanization activity	15	Hemolymph from a single larva was defined as one independent biological replicate. A total of 15 larvae were randomly selected from four independent silk webs.	None
Cellular immunity assays	Encapsulation activity	30	One gel bead co-incubated with larval hemolymph was regarded as one independent replicate. Samples were obtained from four independent silk webs, with five larvae randomly selected from each web.	None
